# Antifungal peptides from living organisms

**DOI:** 10.3389/fmicb.2024.1511461

**Published:** 2024-12-17

**Authors:** Yi Gong, Qunhang Xue, Jun Li, Shicui Zhang

**Affiliations:** ^1^Shanxi Key Laboratory of Birth Defect and Cell Regeneration, MOE Key Laboratory of Coal Environmental Pathogenicity and Prevention, Department of Biochemistry and Molecular Biology, Shanxi Medical University, Taiyuan, China; ^2^Key Laboratory of Biological Resources and Ecology of Pamirs Plateau in Xinjiang Uygur Autonomous Region, College of Life and Geographic Sciences, Kashi University, Kashi, China; ^3^Department of Marine Biology, Institute of Evolution and Marine Biodiversity, Ocean University of China, Qingdao, China; ^4^Laboratory for Marine Biology and Biotechnology, Pilot National Laboratory for Marine Science and Technology, Qingdao, China

**Keywords:** antifungal peptide, sources, mechanisms, production, application

## Abstract

In the post-COVID-19 era, people are increasingly concerned about microbial infections, including fungal infections that have risen in recent years. However, the currently available antifungal agents are rather limited. Worse still, the widespread use of the antifungal agents has caused the emergence of antifungal resistance in *Candida*, *Cryptococcus*, and *Aspergillus* species. Therefore, the development of novel antifungals is urgently needed. Antimicrobial peptides (AMPs), as components of the first-line defense of the host, are found to exhibit broad antimicrobial activity against bacteria, fungi, parasites, viruses, and protozoa. AMPs with antifungal activity are specifically referred to as antifungal peptides (AFPs). AFPs are currently regarded as the most promising alternative to conventional antifungal agents due to the fact that they are highly selective and less prone to facilitate the selection of drug resistance. In this review, we present an overview of the origin and classification of natural AFPs as well as their modes of action. Additionally, the production of natural, semisynthetic, and synthetic AFPs with a view to greater levels of exploitation is discussed. Finally, we evaluate the current and potential applications of AFPs in clinics and in the food industry.

## 1 Introduction

Fungi are eukaryotic microorganisms ranging from giant mushrooms to tiny multicellular molds and unicellular yeasts. It is estimated that there are approximately 2–11 million fungi on Earth, of which only 150,600 are officially categorized (Phukhamsakda et al., 2022; Lücking et al., 2021; Baldrian et al., 2021). Fungi are widely distributed in the soil and the air, in lakes, rivers, and oceans, on plants and animals, and in food and clothing. In recent years, fungi have been found to be a part of the commensal microbiota at different sites of human bodies (e.g., oral cavity, intestine, skin, lung, and vagina), although it remains still controversial over what constitutes the standard mycobiome composition (Auchtung et al., 2018; Huffnagle and Noverr, 2013; Kapitan et al., 2018).

Fungi are beneficial to many aspects of our daily life, notably the production of bread, wine, beer, soy sauce, and certain cheeses. Fungi are also used as a source of food; for example, some mushrooms, morels, and truffles are epicurean delicacies (Campbell-Platt and Cook, 2008; Money, 2016; Mukherjee et al., 2018). However, fungi have a harmful side too. It has been reported that at least 300 species of fungi can cause infections in both human beings and animals (Gupta et al., 2017). Recently, a list of fungal priority pathogens has been presented by the World Health Organization (2022) to guide research, development, and public health action. The list includes 19 fungal pathogens that are ranked and categorized into three priority (critical, high, and medium priority) groups based on their mortality rate, infection rate, and difficulty in diagnosis and treatment. The critical group includes *Candida albicans*, *Aspergillus fumigatus*, *Candida auris,* and *Cryptococcus neoformans*; the high group contains *Candida glabrata*, *Candida parapsilosis*, *Candida tropicalis*, *Fusarium* spp., *Histoplasma* spp., Mucorales, and eumycetoma causative agents; and the medium group comprises *Candida krusei*, *Pneumocystis jirovecii*, *Scedosporium* spp., *Cryptococcus gattii*, *Lomentospora prolificans*, *Coccidioides* spp., *Talaromyces marneffei,* and *Paracoccidioides* spp. Fungal infections have become a serious threat to human health, especially for people with weakened immune systems and potential health problems, such as diabetes mellitus, cancer, and HIV. It is estimated that fungal infections annually affect approximately 25% of the general population globally, causing high morbidity and mortality rates (Brown et al., 2012; Gamaletsou et al., 2018). Unfortunately, there are limited effective antifungal agents to treat fungal infections (Kathiravan et al., 2012). Currently, only four classes of antifungal drugs, i.e., polyenes (e.g., amphotericin B), triazoles (e.g., fluconazole), echinocandins (e.g., caspofungin), and fluorinated pyrimidines (e.g., 5-flucytosine), are available for the choice of systemic therapy of fungal diseases, and most of them, especially amphotericin B, can induce nephrotoxicity and hematotoxicity (Wang et al., 2024; Turcu et al., 2009). Worse still, the restricted spectrum and widespread use of the antifungal agents have caused the emergence of antifungal resistance in *Candida*, *Cryptococcus*, and *Aspergillus* species (Fisher et al., 2018; Lestrade et al., 2019; Pfaller et al., 2019). Furthermore, some fungal pathogens, such as *Mucorales*, *C. auris*, and some molds, are intrinsically resistant to the drugs above and difficult to treat at present. These all prompt an urgent need for the development of new antifungal agents with high efficiency and low toxicity. Of note, fungal cells are eukaryotic, and the development of selective antifungals is thus a particularly great challenge to identify pathogen-specific targets that are not present in human cells.

In addition to infection of humans and animals, fungi can also cause food spoilage, which leads to economic losses and may affect human health. Food-spoiling fungi and their mycotoxins released contaminate approximately 25% of raw materials produced by agriculture worldwide (World Health Organization (WHO), 1999). Therefore, the control and prevention of fungal pathogens and foodborne poisoning is one of the most important public health challenges that we are facing today. A number of physical, chemical, and biological methods have been applied to control fungal pathogens and mycotoxin contamination, including green and emerging technologies such as ionizing and non-ionizing radiation, ultrasound, pulsed electric field and high-pressure processing, and biological preservation. Among them, the use of antifungal compounds is regarded as an alternative environmentally friendly strategy. However, the antifungal compounds currently available for this use are quite limited (Thery et al., 2019). Antimicrobial peptides (AMPs) can really be a good candidate, given their lower likelihood of selecting resistance.

AMPs, first described by Dubos (1939) from *Bacillus brevis*, are short peptides with rapid microbicidal effects that are typically composed of <100 amino acids. Albeit highly diverse in amino acid sequence, AMPs usually possess a net positive charge and hydrophobic regions and facilitate interactions with membranes. AMPs kill microbes via several mechanisms, including binding to or inserting into microbial membranes (which has fatal depolarization of the normally polarized membrane), forming physical pores, disrupting the usual distribution of lipids between the bilayer leaflets, and damaging critical intracellular targets (Gennaro and Zanetti, 2000; Hancock, 2000; Nawrocki et al., 2014). Because of their multi-point and multi-level mechanisms of action, the likelihood of developing resistance to AMPs is relatively low.

AMPs, as components of the first-line defense of the host, are produced by all organisms, from bacteria to humans (Faruck et al., 2016; Kang et al., 2015; Shishido et al., 2015; Silva et al., 2014; Tam et al., 2015), and exhibit broad antimicrobial activity against bacteria, fungi, parasites, viruses, and protozoa (Hancock and Chapple, 1999). Currently, there are 1,479 peptides with antifungal properties documented in the Antimicrobial Peptide Database (APD3). In the majority of cases, the classification of AFPs is based on the peptide origin: natural, semisynthetic, or synthetic (De Lucca, 2000). In this study, we present an overview of the origin and classification of natural AFPs and their modes of action. In addition, the production of natural, semisynthetic, and synthetic AFPs with a view to greater levels of exploitation is discussed. Finally, we evaluate the current and potential applications of AFPs in clinics and in the food industry.

## 2 Origin and classification of AFPs

The innate immunological components including endogenic peptides of organisms could rapidly respond to invading pathogens to avoid their adverse effects on the host. Natural AFPs are produced by a number of species of bacteria, archaea, and eukarya isolated from natural sources (De Lucca and Walsh, 1999). They typically adopt an *α*-helix structure, *β*-hairpin or sheet (containing two cysteine residues) structure, or mixed α-helix/β-sheet structure upon interaction with membranes. Some natural AFPs are rich in specific amino acids such as glycine, proline, arginine, histidine, and tryptophan, and accordingly, they are often classified as glycine-rich, proline-rich, arginine-rich, histidine-rich, and tryptophan-rich AFPs (Bondaryk et al., 2017).

### 2.1 AFPs from microorganisms

The AFPs produced by microorganisms, including bacteria and archaea (both prokaryotes) as well as fungi (eukaryotes), can be secreted into extracellular surroundings and offer a competitive advantage in ecological niches. Bacteria generate a number of different AFPs (Table 1). The first example of an archaeal antimicrobial peptide with antifungal activity is VLL-28, isolated from the archaeon *Sulfolobus islandicus*, which showed antifungal activity against 10 clinical isolates of *Candida* spp. (Roscetto et al., 2018). The well-known AFP-producing bacteria include the genera *Bacillus*, *Lactobacillus*, *Streptomyces*, and *Burkholderia*. For example, the iturin A produced by *Bacillus subtilis* exhibits a conspicuous antifungal activity against *Aspergillus* spp., *Fusarium* spp., and *Penicillium* spp. (Klich et al., 1991); the peptide mixture generated by *Lactobacillus plantarum* TE10 suppresses *Aspergillus flavus* in maize seeds, displaying a considerable potential for the development of bio-control agents (Muhialdin et al., 2020); the champacyclin, a head-to-tail cyclic octapeptide obtained from *Streptomyces champavatii*, inhibits the growth of the yeast *C. glabrata* (Pesic et al., 2013); the natamycin from *Streptomyces philanthi* RL-1-178 possesses a fungicidal activity against *A. flavus* (Boukaew et al., 2023); and the AFC-BC11, a lipopeptide isolated from *Burkholderia cepacia*, exerts an antifungal activity toward a variety of soil fungi (Kang et al., 1998). Similarly, eukaryotic microorganisms such as filamentous fungi and yeasts also produce a variety of AFPs (Table 1). For instance, the peptide PeAfpC produced by the filamentous fungus *Penicillium expansum* can effectively inhibit the growth of *Byssochlamys spectabilis*, which is capable of causing the spoilage of pasteurized juices and canned foods (van der Weerden et al., 2013). In addition, the two peptides, namely, PAF and PAFB, generated by the filamentous fungus *Penicillium chrysogenum*, are capable of exerting antifungal activity against a variety of filamentous fungi, with PAFB suppressing the growth of some toxigenic molds (Kaiserer et al., 2003; Rodríguez-Martín et al., 2010; Huber et al., 2018).

**Table 1:** Representative antifungal peptides from microorganisms and plants.

Organisms	Peptide (length)	Net charge; hydrophobic residue %	Origin	Structure	Antifungal spectrum	References
Archaea	VLL-28 (28)	+10; 35%	*Sulfolobus islandicus*	Helix	*C. albicans, C. parapsilosis, C. tropicalis, C. glabrata, C. krusei*	Roscetto et al. (2018) and Notomista et al. (2015)
Bacteria	Iturin A	–	*Bacillus subtilis*	cyclic peptidolipid	*Aspergillus* spp.*, Fusarium* spp.*, and Penicillium* spp.	Klich et al. (1991)
	Champacyclin	–	*Streptomyces champavatii*	cyclic octapeptide	*C. glabrata*	Pesic et al. (2013)
	Natamycin	–	*Streptomyces philanthi* RL-1-178	–	*A. flavus*	Boukaew et al. (2023)
	AFC-BC11	–	*Burkholderia cepacia*	–	Variety of soil fungi	Kang et al. (1998)
	AFP1 (87)	−3; 36%	*Streptomyces tendae* Tu901	Beta	*Aspergillus* spp.	Bormann et al. (1999)
Fungi	PeAfpC		*Penicillium expansum*		*B. spectabilis*	van der Weerden et al. (2013)
	Antifungal peptide (AgAFP) (51)	+9; 31%	*Aspergillus giganteus*	Beta	*Fusarium* spp.	Theis et al. (2005)
	PAF (55)	+5; 25%	*Penicillium chrysogenum*	Beta	Filamentous fungi *A. flavus, A. fumigatus, A. giganteus, A. niger, B. cinerea, C. carbonum, F. oxysporum, G. roseum, M. circinelloides, N. crassa, P. chrysogenum, and T. koningii*	Kaiserer et al. (2003)
	PAFB (PgAFP) (58)	+4; 27%	*Penicillium chrysogenum* RP42C/Q176	Beta	Filamentous fungi *A. fumigatus, A. niger, A. terreus, N. crassa, P. chrysogenum, T. rubrum,* yeasts *C. albicans, and S. cerevisiae*	Rodríguez-Martín et al. (2010) and Huber et al. (2018)
Plants	*Chitinases*					
	PR protein families	–	–	–	*B. cinerea*	Van Baarlen et al. (2007)
	Chitinase	–	–	–	*R. solani*	Shrestha et al. (2007)
	*Defensins*					
	Dm-AMP1 (50)	+1; 38%	*Dahlia merckii*	Combined Helix/Beta	*B. cinerea, C. sphaerospermum, F. culmorum, L. maculans, P. digitatum, S. tritici, and V. albo-atrum*	Osborn et al. (1995)
	Ace-AMP1		*Allium cepa*	Combined Helix/Beta	*A. solani, F. solani, and F. oxysporum*	Wu et al. (2011) and Tassin et al. (1998)
	MsDef1 (45)	+3; 33%	*Medicago sativa*	Combined Helix/Beta	*V. Dahliae, F. graminearum, A. solani*., and *F. culmorum*	Gao et al. (2000)
	MtDef4 (47)	+6; 31%	*Medicago truncatula*	Combined Helix/Beta	*F. graminearum*	Ramamoorthy et al. (2007)
	RsAFP2 (51)	+6; 39%	*Raphanus sativus*	Combined Helix/Beta	*B. cinerea, C. sphaerospermum, F. culmorum, L. maculans, P. digitatum, T. viride, S. tritici*, *V. albo-atrum, C. albicans, P. pastoris, C. krusei, A. flavus, F. solani, and F. graminearum*	Osborn et al. (1995) and Terras et al. (1993)
	PvD1 (21)	−1; 21%	*Phaseolus vulgaris*	Bridge	*C. albicans, C. parapsilosis, C. tropicalis, C. guilliermondii, K. marxiannus, S. cerevisiae, F. oxysporum, F. solani, F. lateritium, and R. solani*	Games et al. (2008) and Mello et al. (2011)
	Hevein-type					
	Ee-CBP (45)	+5; 28%	*Euonymus europaeus* L.	Bridge	*A. brassicicola, B. cinerea, F*. *culmorum, F. oxysporum* f.sp. *cubense*, *F. oxysporum* f.sp. *matthiolae*, *M. eumusae, N. crassa, P. exigua, P. cryptogea, P. ultimum, R. solani, and T. hamatum*	Van den Bergh et al. (2002)
	SmAMP3 (35)	+2; 34%	*Stellaria media* L.	Bridge	*A. niger, B. sorokiniana, B. cinerea, F. solani, and A. alternata*	Rogozhin et al. (2015)
	WAMP-1a (44)	+3; 38%	*Triticum kiharae*	Combined Helix/Beta	*B. sorokiniana, B. cinerea, N. crassa, F. oxysporum, F. verticillioides, and F. solani*	Odintsova et al. (2009)
	Snakins					
	Snakin-1 (63)	+8; 31%	*Solanum tuberosum*	Helix	*B. cinerea, F. solani, F. culmorum, F. oxysporum* f. sp. *conglutinans, F. oxysporum* f. sp. *lycopersici, P. cucumerina, C. graminicola, C. lagenarium, B. maydis, and A. flavus*	Segura et al. (1999)
	Snakin-2 (66)	+9; 34%	*Solanum tuberosum* cv. Jaerla	Bridge	*B. cinerea, F. solani, F. culmorum, F. oxysporum* f. sp. *conglutinans, F. oxysporum* f. sp. *lycopersici, P. cucumerina, C. graminicola, C. lagenarium, B. maydis, and A. flavus*	Berrocal-Lobo et al. (2002)
	Gly-rich peptides					
	Cc-GRP (35)	−1; 0%	*Coffea canephora*	Gly-rich	*F. Oxysporum and C. lindemuthianum*	Zottich et al. (2013)

### 2.2 AFPs from plants

Plants have developed various mechanisms in their innate immune systems to protect themselves against fungal attacks, including soluble peptides and proteins released from plants with antifungal activities (Chiu et al., 2022). These peptides/proteins that are constitutively synthesized are able to trigger defense responses in plants. On the basis of sequence, cysteine residues, and function, the plant-sourced AFPs can be divided into different families (Table 1), including chitinases, defensins, and snakins, as well as hevein-type and gly-rich peptides (Tam et al., 2015; Yan et al., 2015). Chitinases are among the best-known and most-studied plant AFPs. They display strong antifungal activity against a wide range of phytopathogenic fungi, including *Botrytis cinerea* (Van Baarlen et al., 2007) and *Rhizoctonia solani* (Shrestha et al., 2007). Plant chitinases have been used to treat fungal infections as exogenously applied pest control agents (Karasuda et al., 2003). The Dm-AMP1 is a defensin peptide found in *Dahlia merckii* that shows inhibitory activity against a variety of fungi, such as *B. cinerea* and *Leptosphaeria maculans* in the presence of CaCl_2_ and KCl (Osborn et al., 1995). Another defensin Ace-AMP1, a potent AFP found in onion (*Allium cepa*) seeds, has been applied to control the tomato early blight disease caused by the pathogen *Alternaria solani* (Wu et al., 2011). The peptide Ee-CBP containing five disulfide bridges obtained from the bark of spindle tree (*Euonymus europaeus*) is a hevein-type AFP, exhibiting antifungal activity against various fungi including *Alternaria brassicicola* (50% growth inhibition IC_50_ = 3 μg/mL), *Phoma exigua* (IC_50_ = 33 μg/mL), and *Fusarium oxysporum* f.sp. *cubense* (IC_50_ = 15 μg/mL) (Van den Bergh et al., 2002). The WAMP-1a, another hevein-type AFP from seeds of *Triticum kiharae*, shows high broad-spectrum inhibitory activity against a wide range of chitin-containing and non-chitin-containing pathogens including *Bipolaris sorokiniana*, *Neurospora crassa*, *Fusarium verticillioides,* and *Fusarium solani* (Odintsova et al., 2009). The snakins 1 and 2 are representative AFPs of snakin family identified from *Solanum tuberosum*, exerting antifungal activity against fungi such as *B. cinerea*, *F. solani*, *A. flavus*, *Colletotrichum graminicola*, and *Bipolaris maydis* (Segura et al., 1999; Berrocal-Lobo et al., 2002). The Cc-GRP is a gly-rich AFP identified from *Coffea canephora* that can combat fungi such as *F. oxysporum* and *Colletotrichum lindemuthianum* (Zottich et al., 2013).

### 2.3 AFPs from animals

Many animal-sourced AFPs have been found to be part of the innate immune responses of both invertebrates and vertebrates (Tables 2, 3). As invertebrates lack adaptive immunity, AMPs play a significant role in their immune response and comprise an essential source of AFPs (Table 2). The AFPs obtained from marine invertebrates include penaeidins, Cm-p1, and tachystatins. The penaeidin family originates from shrimp, and currently, there are nine members available for this family in the ADP3 database (Destoumieux et al., 2000; Cuthbertson et al., 2002; An et al., 2016). The members of the penaeidin family show broad-spectrum fungicidal activity. For example, both penaeidins 2 and 3a are fungicidal against filamentous fungi and shrimp pathogen *F. oxysporum* (Destoumieux et al., 2000). Cm-p1 is a 10-mer short peptide isolated from marine snails. Cm-P1 has the ability to inhibit the growth of yeasts and filamentous fungi, while it shows little toxic effects on mammalian cells (López-Abarrategui et al., 2012). The tachystatin family consists of four members: tachystatin A, tachystatin B1, tachystatin B2, and tachystatin C, all of which have been identified in horseshoe crabs. All four members of the tachystatin family contain three disulfide bridges and have sequences similar to spider neurotoxins. Since horseshoe crabs are close relatives of spiders, tachystatins and neurotoxins may have evolved from a common ancestral peptide gene. Tachystatins are capable of binding to chitin and then exert their antifungal activity against *C. albicans* and *Pichia pastoris* (Osaki et al., 1999). AFPs are also found in insects. Representative AFPs from insects include melittin and thanatin. Melittin was isolated from bee venom by Neuman et al. (1952), which existed in hemolysin phospholipase A (Habermann, 1972). Melittin shows strong antifungal activity against various strains of fungi, including *Aspergillus* sp., *Candida* sp., *Malassezia* sp., *Penicillium* sp., and *Trichoderma* sp. (Memariani and Memariani, 2020). It is found that melittin exerts inhibitory activity against fungi via a series of combined mechanisms of inhibition of (1,3)-*β*-D-glucan synthase, membrane permeabilization, apoptosis induction by reactive oxygen species (ROS), and alterations in gene expression (Memariani and Memariani, 2020). Thanatin, a 21-residue peptide, was first isolated from the insect *Podisus maculiventris* (Fehlbaum et al., 1996). It is highly potent in inhibiting the growth of fungi at considerably low concentrations (Fehlbaum et al., 1996; Dash and Bhattacharjya, 2021). Protonectin was originally isolated from the venom of the neotropical social wasp *Agelaia pallipes pallipes* (Mendes et al., 2004). Later, protonectin was shown to have potent antifungal and fungicidal activity against *C. glabrata*, *C. albicans*, *C. parapsilosis*, *C. tropicalis*, and *C. krusei* by disturbing membrane integrity and inducing ROS production in yeast cells (Wang et al., 2015). Recently, a 41-amino acid peptide called blapstin, isolated from the Chinese medicinal beetle *Blaps rhynchopetera,* was shown to possess antifungal activity against *C. albicans* and *Trichophyton rubrum*. Cryo-scanning electron microscope (Cryo-SEM) observations showed that blapstin directly resulted in disruption in the cell structure of *C. albicans* and *T. rubrum* (Zhang et al., 2023).

**Table 2:** Representative antifungal peptides from invertebrates.

	Peptide (length)	Net charge; hydrophobic residue %	Origin	Structure	Antifungal spectrum	References
Marine invertebrate	Penaeidins family	–	*Penaeus vannamei*	Helix/random coil	Filamentous fungi; shrimp pathogen *F. oxysporum*	Destoumieux et al. (2000)
	Cm-p1 (10)	+1; 30%	*Cenchritis muricatus*	Helix	*A. niger, C. albicans* 01 U, *C. albicans* 38 U, *C. parapsilosis, C. neoformans*, and *T. rubrum*	López-Abarrategui et al. (2012)
	Polyphemusin I, II (18)	+8; 44%	*Limulus polyphemus*	Beta	*C. albicans* M9	Miyata et al. (1989)
	Tachyplesin II (17)	+7; 47%	*Tachypleus tridentatus*	Beta	*C. albicans* M9	
	Big defensin (79)	+6; 45%		Combined Helix/Beta	Fungi such as *C. albicans*	Saito et al. (1995)
	Tachystatins family	–	*Tachypleus tridentatus; Limulus polyphemus*	Beta	*C. albicans and P. pastoris*	Osaki et al. (1999)
	Arenicin-1 (21)	+6; 52%	*Arenicola marina*	Beta	*C. albicans* 820	Ovchinnikova et al. (2004)
Insect	Lasiocepsin (27)	+9; 48%	*Lasioglossum laticeps*	Helix	*C. albicans*	Monincová et al. (2012)
	Melittin (26)	+6; 46%	*Apis mellifera*	Helix	*C. albicans*, *T. beigelii*, and *M. furfur*	Fennell et al. (1967) and Sung et al. (2008)
	Papiliocin (37)	+8; 48%	*Papilio xuthus*	Helix	Yeast	Kim et al. (2010)
	Polybia-CP (12)	+2; 58%	*Polybia paulista*	Helix	Yeast	Souza et al. (2005)
	Protonectin (12)	+2; 58%	*Agelaia pallipes pallipes*	Helix	*Candida* spp.	Mendes et al. (2004)
	Spinigerin (25)	+5; 52%	*Pseudacanthotermes spiniger*	Helix	Various filamentous fungi and yeast strains	Lamberty et al. (2001b)
	Termicin (36)	+6; 50%	*Pseudocanthothermes* *spiniger, Reticulitermes flavipes*	Combined Helix/ Beta	*F. Culmorum, F. oxysporum, N. crassa, N. haematococca, and T. viride*	Lamberty et al. (2001b)
	Heliomicin (44)	+2; 36%	*Heliothis virescens*	Combine Helix/ Beta	*C. albicans and P. pastoris*	Lamberty et al. (2001a)
	Thanatin (21)	+6; 28%	*Podisus maculiventris*	Beta	*N. crassa, N. crassa, N. haematococca, T. viride, A. brassicicola, F. culmorum, A. pisi, and F. oxysporum*	Fehlbaum et al. (1996) and Dash and Bhattacharjya (2021)
	Alo-3 (36)	+5; 27%	*Acrocinus longimanus*	Beta	*C. albicans and C. glabrata*	Barbault et al. (2003)
	Psacotheasin (34)	+2; 35%	*Psacothea hilaris*	Knottin-type	*C. albicans*	Hwang et al. (2010)
	ARD1 (41)	+3; 39%	*Archeoprepona demophoon*	Combined Helix/ Beta	*Fumigatus and C. albicans*	Landon et al. (2004)
	Coprisin (43)	+3; 51%	*Copris tripartitus*	Combined Helix/ Beta	*A. flavus, A. fumigatus, A. parasiticus, C. albicans, C. parapsilosis, M. furfur, T. beigelii, and T. rubrum*	Hwang et al. (2009) and Lee et al. (2012)
	Drosomycin (44)	+1; 34%	*Drosophila melanogaster*	Combined Helix/ Beta	Filamentous fungi: *A. fumigatus*, *A. ustus*, *F. solani*, and *F. oxysporum*	Fehlbaum et al. (1994) and Simon et al. (2008)
	Gambicin (61)	+4; 39%	*Anopheles gambiae*	Beta	Filamentous fungus *N. crassa*	Vizioli et al. (2001)
	Es-termicin (35)	+1; 45%	*Eupolyphaga sinensis*	Combined Helix/ Beta	*C. albicans*	Liu et al. (2016)
	Blapstin (41)	+4; 27%	*Blaps rhynchopetera*	Bridge	*C. albicans and T. rubrum*	Zhang et al. (2023)
Other Arthropoda	Juruin (38)	+5; 42%	*Avicularia juruensis*	Cystine-knot	*C. albicans, C. krusei, C. glabrata, C. parapsilosis, C. tropicalis, C. guilliermondii, and A. niger*	Ayroza et al. (2012)
	Gomesin (18)	+6; 33%	*Acanthoscurria gomesiana*	Beta	Fungi *A. brassicicola, A. fumigatus, F. culmorum, F. oxysporum, N. crassa, N. haematococca, T. viride, T. mentagrophytes*; yeasts *C. albicans, C. tropicalis, C. neoformans, S. cerevisiae, C. glabrata*, and *B. bassiana*	Silva et al. (2000)
	LBLP (23)	+8; 17%	*Scolopendra subspinipes mutilans*	Helix/random coil	*C. albicans, C. parapsilosis, M. furfur, and T. beigelii*	Choi et al. (2013)

**Table 3:** Representative antifungal peptides from vertebrates.

	Peptide (length)	Net charge; hydrophobic residue %	Origin	Structure	Antifungal spectrum	References
Fish and Amphibian	Piscidins family	–	Various fish taxa	Helix	*C. albicans, M. furfur, and T. beigelii*	Asensio-Calavia et al. (2023) and Rakers et al. (2013)
	Pleurocidin (25)	+4; 44%	*Pleuronectes americanus*	Helix	*C. albicans, F. oxysporum, A. niger, and Alternaria* spp.	Cole et al. (1997) and Souza et al. (2013)
	Misgurin (21)	+7; 28%	*Misgurnus anguillicaudatus*	Helix	*C. albicans, C. neoformans, and S. cerevisiae*	Park et al. (1997)
	Magainin 2 (23)	+3; 43%	*Xenopus laevis*	Helix	*C. albicans and S. cerevisiae*	Zasloff (1987)
	PGLa (21)	+5; 61%	*Xenopus laevis*	Helix	*C. albicans and S. cerevisiae*	Soravia et al. (1988)
	Temporins G (13)	+2; 61%	*Rana temporaria*	–	*Candida* spp.*, C. neoformans*, and *Aspergillus* spp.	Simmaco et al. (1996) and D'Auria et al. (2022)
	Andricin B (10)	+2; 50%	*Andrias davidianus*	Random coil	*A. niger, C. albicans, and S. cerevisiae*	Pei et al. (2018)
Mammal	Defensins					
	HNP-1, HNP-2, HNP-3, HNP-4	–	*Homo sapiens*	Beta	*C. albicans*	Selsted et al. (1985), Lehrer et al. (1988), and Wilde et al. (1989)
	NP-1 (33)	+9; 51%	*Oryctolagus cuniculus*	Bridge	*C. neoformans*	Alcouloumre et al. (1993)
	hBD2 (41)	+7; 36%	*Homo sapiens*	Combined Helix/ Beta	*Candida* spp.	Joly et al. (2004)
	RTD-1 (18)	+5; 55%	*Rhesus Macaque*	Beta	*C. albicans and C. neoformans*	Tran et al. (2002)
	Cathelicidins					
	SMAP-29 (29)	+10; 37%	*Ovis aries*	Helix	*C. albicans, C. neoformans, and R. rubra*	Skerlavaj et al. (1999)
	Indolicidin (13)	+4; 53%	*Bos taurus*	Extended boat-shaped structure	*C. albicans, S. cerevisiae, and T. beigelii*	Lee et al. (2003)
	LL-37 (37)	+6; 35%	*Homo sapiens; Pan troglodytes*	Helix	*Candida* spp.	Scarsini et al. (2015)
	Histatins					
	Human Histatins 1–9	–	*Homo sapiens*	His-rich classic	Histatin 5: *C. albicans, C. glabrata, C. krusei, C. neoformans, and S. cerevisiae*	Sharma et al. (2021)
	Mfa-hst5 (30)	+10; 3%	*Macaca fascicularis*	Helix	*C. albicans, C. neoformans, C. lusitaniae, and C. tropicalis*	Padovan et al. (2010)
	Lactoferricins					
	Lactoferricin B (25)	+8; 48%	*Bos taurus*	Beta	*A. fumigatus, F. solani, and C. albicans*	Sengupta et al. (2012)

Spiders and centipedes are also known to produce AFPs, such as juruin, gomesin, and lactoferricin B-like peptide (LBLP), especially in venoms. Juruin isolated from the venom of the Amazonian pink toe spider *Avicularia juruensis* has antifungal activity against filamentous fungi and yeasts, including *Aspergillus niger*, *Beauveria bassiana*, *C. albicans*, *C. krusei*, *C. glabrata*, *C. parapsilosis*, *C. tropicalis,* and *Candida guilliermondii* (Ayroza et al., 2012). Gomesin, an 18-amino acid AMP isolated from the hemolymph of the tarantula spider *Acanthoscurria gomesiana*, inhibits the development of filamentous fungus and yeast (Silva et al., 2000). LBLP, a 23-mer AMP derived from the centipede *Scolopendra subspinipes mutilans*, has been found to have antifungal and fungicidal activity against *C. albicans*, *C. parapsilosis*, *Malassezia furfur*, and *Trichosporon beigelii* by forming pores in the membrane, eventually leading to fungal cell death (Choi et al., 2013). Recently, LBLP has been reported to trigger mitochondrial disruption-mediated apoptosis by inhibiting respiration under nitric oxide accumulation in *C. albicans* (Kim et al., 2020).

In vertebrates (Table 3), the immune system is divided into innate immunity and adaptive immunity. Adaptive immunity provides an effective and specific immune response against pathogens, while innate immunity consisting of the first line of defense is much quicker to respond to initial attacks. AMPs produced in response to pathogenic attacks form part of the first line of defense and are a source of AFPs. AFPs derived from vertebrates are usually produced on the skin, mucous membranes, and other areas that are easily exposed to microbial environments (López-Meza et al., 2011). It has been shown that the piscidins synthesized in the epithelia of gills, skin, stomach, and gut of a variety of teleost species exhibit antifungal activity against *C. albicans*, *M. furfur,* and *T. beigelii* through membrane disruption mode (Asensio-Calavia et al., 2023; Rakers et al., 2013). In addition, pleurocidin secreted by the skin of winter flounder inhibits the growth of *Alternaria* spp., *C. albicans*, *F. Oxysporum*, and *A. niger* (Cole et al., 1997; Souza et al., 2013). Recently, we have shown that AP10W, a short peptide derived from AP-2 complex subunit mu-A of zebrafish, displays conspicuous antifungal activities against the main fungal pathogens of human infections *C. albicans* and *A. fumigatus*. We also show that AP10W inhibits fungal biofilm formation and decrease pre-established fungal biofilms (Gong et al., 2022).

Similarly, the skin and secretory glands of amphibian frogs are also a rich source of AFPs, such as magainins and peptide glycine-leucine-amide (PGLa) identified in the clawed frog *Xenopus laevis* (Zasloff, 1987; Soravia et al., 1988), and temporins A, B, and L identified in the red frog *Rana temporaria* (Simmaco et al., 1996; Marcocci et al., 2018; Roscetto et al., 2021). Temporin G, recently isolated from the skin of *Rana temporaria*, is demonstrated to exert fungicidal ability against *C. neoformans, Candida* spp., and *Aspergillus* spp. In addition, temporin G reduces the metabolic activity of *C. albicans* cells, induces moderate membrane perturbation, and is effective against virulence factors of *C. albicans* (D'Auria et al., 2022).

In mammals, both neutrophils and epithelial cells are known to produce AFPs, including defensins, cathelicidins, histatins, and lactoferricins. Defensins are widely present in eukaryotes (fungi, plants, and animals), with four types of human defensins, known as HNP-1, HNP-2, HNP − 3, and HNP-4. All human defensins have been shown to possess candidacidal ability and are capable of influencing the ionic environment and the metabolic state of *C. albicans* cells (Selsted et al., 1985; Lehrer et al., 1988; Wilde et al., 1989). Cathelicidins have been identified in both humans and chimpanzees. LL-37, a 37-mer peptide derived from the N-terminal 37 residues of human cathelicidins, inhibits the growth of fungi including *Aspergillus*, *Candida*, *Colletotrichum*, *Fusarium*, *Malassezia*, *Pythium*, and *Trichophyton*. LL-37 exerts its fungal inhibition through several mechanisms, including cell wall integrity disruption, membrane permeabilization, and intracellular effects such as formation of autophagy-like structures, disturbance of endoplasmic reticulum homeostasis, induction of oxidative stress, inhibition of cell cycle progression, and alterations in gene expression (Memariani and Memariani, 2023). Histatins are histidine-rich peptides abundantly present in human saliva and the oral cavity (Oppenheim et al., 1988). Histatins exhibit a broad spectrum of antifungal activities and play an important role in controlling periodontal and oral fungal infections (Kavanagh and Dowd, 2004; Pólvora et al., 2018). Human histatins are composed of nine classes (human histatins 1–9), and histatin 5 is found to have strong fungicidal activity against *C. albicans*, *C. glabrata*, *C. krusei*, *C. neoformans,* and *Saccharomyces cerevisiae* (Sharma et al., 2021). Lactoferrin is a glycoprotein with AMP activity, found in saliva, milk, vaginal secretions, tears, and other exocrine secretions of mammals (cows, pigs, mice, and humans) (Moreno-Expósito et al., 2018; Rascón-Cruz et al., 2021; Drago-Serrano et al., 2018). Lactoferricin is found to have antifungal activity against a variety of fungi including *Clavispora lusitaniae*, *Pichia kudriavzevii*, *Kluyveromyces marxianus*, *Meyerozyma guilliermondii*, *S. cerevisiae*, *Candida* spp., and *Cryptococcus* spp.(Fernandes et al., 2020). Moreover, lactoferricin also shows inhibitory activity against the fungal biofilm formed by keratitis-associated fungal pathogens, such as *A. fumigatus*, *F. solani*, and *C. albicans* (Sengupta et al., 2012).

## 3 Modes of action of AFPs

**Figure 1:**
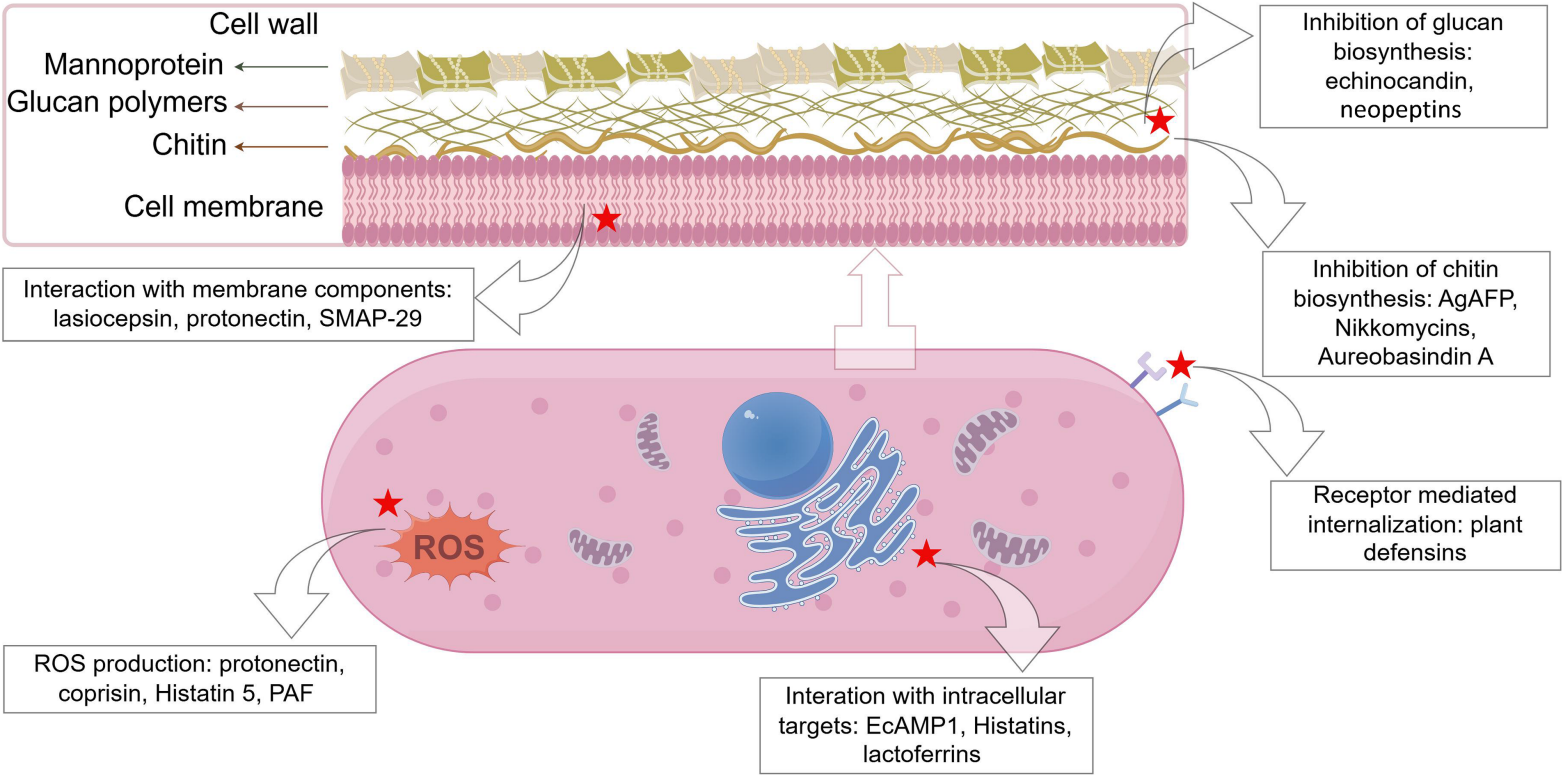
Schematic representation of the targets of some representative antifungal peptides. The red star marks the targets of AFPs (created by figdraw).

Exploration of the modes of action of AFPs is a significant aspect of AFP research as the understanding of antifungal mechanisms can be a great help for researchers to further develop and design novel AFPs. In general, AFPs are known to function via three modes of action (Figure 1), i.e., inhibition of biosynthesis of cell wall components, interaction with membrane components, and interference with intracellular targets (Zhang et al., 2021). It is notable that some AFPs often function via multiple modes of action. For example, the fish-sourced peptide AP10W has been shown to exert its fungicidal activity through modes of combined actions, including interaction with the fungal cell walls via laminarin, mannan, and chitin, enhancement of cell wall permeabilization, induction of membrane depolarization, and increase in intracellular ROS generation (Gong et al., 2022).

### 3.1 Inhibition of biosynthesis of cell wall components

The fungal cell wall, the first barrier of the cell to effectively resist the influence of the external environment, is mainly composed of chitin, mannans, glycoproteins, and glucans (*β* − 1,3-glucan, β − 1,6-glucan, *α*-1,3-glucan, α-1,4-glucan, and mixed β-1,3−/β-1,4-glucan) (Bowman and Free, 2006). It protects the fungal cell from the pressure of the external environment and maintains normal cell metabolism, ion exchange, and osmotic pressure (Cabib and Arroyo, 2013).

Chitin and β-1,3-glucan are both the major structural components of the cell walls of many fungi and play a key role in maintaining the structural integrity of fungal cell walls (Bowman and Free, 2006; Lenardon et al., 2010; Fleet, 1991). Some AFPs are inhibitors of chitin synthase or (1–3)-β-D-glucan synthase, capable of blocking the synthesis of cell wall components, which then disrupts the normal cell morphology and diminishes the cell’s capacity to regulate osmotic pressure. For example, the fungus-sourced AgAFP suppresses the activity of chitin synthases III and V, which are important for chitin biosynthesis (Hagen et al., 2007). The echinocandin, a cyclic hexapeptide isolated from several species of *Aspergillus*, inhibits β-1,3-D-glucan synthase necessary for glucan biosynthesis (Ciociola et al., 2016; Thorn et al., 2024). Neopeptins have been reported against some plant pathogenic fungi by inhibiting proteoheteroglycan and β-1,3-glucan synthesis, which could affect cell wall biosynthesis (Ubukata et al., 1984). The nikkomycins (a complex of nucleoside-peptides), an analog of chitin synthase natural substrate N-acetylglucosamine, also block the synthesis of chitin in *C. albicans* (McCarthy et al., 1985). Analogously, the aureobasidin A, a cyclic depsipeptide produced by *Aureobasidium pullulans*, interferes with fungal cell wall integrity by affecting actin assembly, chitin delocalization, and synthesis of sphingolipids (Endo et al., 1997; Nagiec et al., 1997).

### 3.2 Interaction with membrane components

AFPs, as part of AMPs, are usually small (< 100 amino acids) cationic polypeptides, with amphiphilic structures with hydrophobic domains capable of binding to lipids and positively charged hydrophilic domains capable of binding to water or negatively charged residues (Hollmann et al., 2018). This property of AFPs renders them able to form strong binding to the amphiphilic part of the cell membrane, which lays the structural basis for the interaction of AFPs with fungal cell membranes. Several models have been proposed to explain the action of membrane disruption caused by AFPs, such as the barrel-stave, toroidal pore, and carpet models (Zhang et al., 2021; Yeaman and Yount, 2003). According to the barrel-stave model, AFPs bind to lipid membranes and recognize each other to form a transmembrane pore (Theis and Stahl, 2004; Soltani et al., 2007). Ultimately, the cell membrane components are penetrated by AFPs, resulting in cell collapse and death. The toroidal model suggests that AFPs insert into the hydrophobic center of the cell membrane (Soltani et al., 2007; Le et al., 2017), triggering the phospholipid molecular layer to curve inward and form a mixed cavity randomly (Yang et al., 2001). As a consequence, the membrane structures are disordered, eventually leading to cell death. In the carpet model, AFPs interact only with the lipid head groups and are oriented parallel to the surface, forming a carpet-like pattern. Once AFPs on the membrane surface accumulate to a certain concentration threshold, they act as detergents by distorting the phospholipid bilayer, reducing the stability of the cell membrane and causing cell membrane disintegration and cell lysis (Lohner and Prenner, 1999; Zhang et al., 2020). For example, plant defensins Dm-AMP1 from dahlia (*Dahlia merckii*), RsAFP2 from radish (*Raphanus sativus*), and HsAFP1 from coral bells (*Heuchera sanguinea*) target specific binding sites on fungal cells (e.g., mannosyl diinositolphosphoryl ceramide from *S. cerevisiae* for Dm-AMP1 and glucosylceramide from *P. pastoris* for RsAFP2), leading to membrane permeabilization and eventually decreasing viability (Thevissen et al., 2003; Thevissen et al., 2004; Aerts et al., 2008). Similarly, protonectin, isolated from the venom of the neotropical social wasp *Agelaia pallipes pallipes*, exerts antifungal/fungicidal activities against the tested fungal cells via interaction with lipid membranes, disruption of the membrane integrity, and induction of the production of intracellular ROS (Wang et al., 2015). MAF-1A, a linear 26-amino acid peptide, is derived from the carboxy-terminal functional domain of the antifungal peptide-1 (MAF-1) isolated from the hemolymph of *Musca domestica* larvae (Zhou et al., 2016). It has been shown that MAF-1A disrupts the cell membrane of *C. albicans* and then enters the cell where it binds and interacts with nucleic acids (Cheng et al., 2021). However, it must be pointed out that although the interaction of AMPs (including AFPs) with biological membranes plays an important role in the entire process of their antimicrobial actions, membrane disruption is a rather complex and dynamic process, which still needs detailed study to refine the specific mechanisms (Haney et al., 2019).

### 3.3 Interference with intracellular targets

AFPs can also interact with fungal intracellular targets, including nucleotides (DNA and RNA), proteins, and organelles. For example, CGA-N9, an antifungal peptide derived from human chromogranin A (CGA), has been found to exert antifungal activity toward *C. tropicalis* by attenuating mitochondrial function (Li et al., 2019). Psd1 defensin from pea (*Pisum sativum*) has been found to enter *N. crassa* cells, localize to the nuclei, and interfere with the cell cycle, probably via interacting with cyclin F (Lobo et al., 2007). Similarly, histatin 5 binds to mitochondria after crossing fungal membranes via transmembrane potentials or receptors (without damaging the plasma membrane) and then induces non-lytic release of adenosine triphosphate (ATP) into the cytoplasm. The released ATP binds to purinergic receptors on the cell surface, leading to the inhibition of mitochondrial respiration and the generation of ROS, which then causes damage to nucleic acids and organelles (Kavanagh and Dowd, 2004; Helmerhorst et al., 2001). The antifungal peptide EcAMP1 isolated from barnyard grass shows strong antifungal action toward species of the *Fusarium* genus. It has been found that EcAMP1 first binds to one or several abundant components of the fungal cell surface and is then internalized by the fungal cell and accumulates in a vesicular structure in the cytoplasm without disturbing the integrity of the membrane (Nolde et al., 2011).

## 4 Production of AFPs

To effectively explore the structure–activity relationships, efficacy, and safety, especially in clinical treatments, it is necessary to produce sufficient amounts of highly pure AFPs. There are currently three major approaches to achieving this goal, i.e., direct isolation from various organisms, recombinant expression, and chemical synthesis (Fernández de Ullivarri et al., 2020).

### 4.1 Natural production

AFPs are naturally isolated from different species of organisms. Currently, only a limited number of AFPs are obtained from their natural sources for clinical use as the isolation of these natural peptides is time-consuming and expensive due to their relatively low abundance (Vriens et al., 2014). The methods commonly used now for industrial-scale AFP production from natural sources are microbial fermentation and proteolysis.

The natural echinocandins, echinocandin B, pneumocandin B0, and FR901379, are produced for commercial purposes from *Aspergillus rugulosus*, *Glarea lozoyensis,* and *Coleophoma empetri*, respectively. The production of echinocandins is an exceptional example of AFPs produced by microbial fermentation and further chemical modification in the case of the semisynthetic variants. The fermentation process is critical to obtain a competitive product and thus needs optimizing to increase the amount of natural echinocandins and control their purification costs. Temperature is a key factor in fermentation production and influences the overall production costs of AFPs (Emri et al., 2013). The optimal temperatures for the production of echinocandin B, pneumocandin B 0, and FR901379 are similar, all below 30°C. However, the optimal growth temperatures of the strains of *A. rugulosus*, *G. lozoyensis*, and *C. empetri* exhibited considerable variation. The optimal growth temperatures of *G. lozoyensis* and *C. empetri* were observed to be lower than 30°C, whereas the optimal growth temperatures of *A. rugulosus* were found to be higher than 30°C, reaching 37°C; it exhibited poor growth at the temperatures utilized for the production of echinocandin B. The addition of complex nitrogen sources and plant oils also influences the generation of echinocandins (Emri et al., 2013).

Production of pharmaceutical-grade AFPs can be achieved through enzymatic hydrolysis of proteins, resulting in the release of encrypted peptides. The process generally involves three steps, i.e., acquisition of raw materials, protein hydrolysis, and fractionation and isolation. By-products from dairy, fish, and meat industries are all suitable sources of proteins (Sibel Akalin, 2014; Ryder et al., 2016). For proteolysis, the utilization of immobilized enzymes possesses several advantages over the conventional soluble enzymes, such as milder and controlled conditions and recycling of enzymes used (Sewczyk et al., 2018). The final step of fractionation and isolation of AFPs includes ultrafiltration, precipitation with solvents, and liquid chromatography techniques, which are usually expensive (Brady et al., 2008). Fortunately, an alternative and cost-effective method, electro-membrane filtration, which combines electrophoresis with conventional membrane filtration, has been established (Bazinet and Firdaous, 2013) and is increasingly being applied for the fractionation and isolation of AFPs.

### 4.2 Recombinant production

Recombinant expression presents a solid option for producing AFPs at low cost and high efficiency. In addition, sequencing technologies have generated a vast amount of genomic and transcriptomic data, providing valuable resources for discovering and designing new and more active AFPs (Amaral et al., 2012; Porto et al., 2012; Tracanna et al., 2017). Bacteria (mainly *Escherichia coli*), yeasts (mainly *P. pastoris*), and plants (e.g., tobacco plant *Nicotiana tabacum*) are the most common expression platforms for recombinant proteins.

*E. coli* BL21 (DE3), deficient in proteases that may lead to protein degradation, is by far the most commonly used bacterial species as a host for recombinant production of proteins (Li, 2011). Many *E. coli* strains are unable to export proteins across their outer membrane. As a result, in the majority of cases, proteins are secreted into the cytoplasm or periplasm, leading to the formation of inclusion bodies (Singh et al., 2015). Examples of AFPs produced in *E. coli* include lactoferricin B, nikkomycin, magainin-2, and cecropin (Fernández de Ullivarri et al., 2020).

In contrast to prokaryotic *E. coli*, eukaryotic yeasts are capable of implementing certain post-translational modifications to heterologous recombinant proteins. Yeasts commonly used as hosts for recombinant proteins include *S. cerevisiae*, *P. pastoris*, *Kluyveromyces lactis*, *Yarrowia lipolytica*, *Schizosaccharomyces pombe*, and *Hansenula polymorpha*. For example, the recombinant antifungal proteins serum albumin and hen lysozyme were produced by *K. lactis* and *S. cerevisiae*, respectively (Vieira Gomes et al., 2018). In addition, filamentous fungi such as *A. pullulans*, *P. Chrysogenum*, and *P. digitatum* have also been used to generate AFPs. Some examples of AFPs produced by yeasts or filamentous fungi are protegrin-1, porcine lactoferrin, aureobasidin A, and NFAP2 (Fernández de Ullivarri et al., 2020; Slightom et al., 2009).

Plants have been explored as hosts for recombinant expression of AFPs due to their capacity for large-scale production and their cost-effectiveness. The advantages of plants as expression systems are their capability to perform appropriate glycosylation, folding, and disulfide bond formation of recombinant AFPs. Different genetic approaches have been employed to produce AMPs in plants including using whole plants, tissue-specific expression, tissue culture, or transient expression (Holaskova et al., 2015). Whole tobacco plants have been utilized to produce lactoferrin and dermaseptin with a higher yield (Chahardoli et al., 2018; Shams et al., 2019).

### 4.3 Chemical synthesis

The chemical synthesis of peptides allows scientists to design and produce specific sequences of AFPs on demand. Ideally, an AFP should be short. *De novo* peptide design may help reduce production costs, potential toxicity, and lability and increase bioactivity *in vivo* (Steckbeck et al., 2014). Peptide chemical synthesis is divided into two types: solid- (SPPS) or liquid (solution)-phase peptide synthesis (LPPS). Currently, fluorenylmethyloxycarbonyl (Fmoc) SPPS is the preferred method for chemical synthesis of AFPs due to the versatility and low cost of very high-quality building blocks. We have recently designed and synthesized a peptide named AP10W that shows increased antifungal activity (Gong et al., 2022).

## 5 Applications and prospects

A primary focus of natural AFP research is the development of novel specific antifungal drugs against fungal infections (Matejuk et al., 2010). A number of AFPs, such as human lactoferricin-based hLF1-11, novexatin, and pexiganan, are in preclinical development, but few of them reach the clinical stage (Ciociola et al., 2016; Duncan and O’Neil, 2013; Koo and Seo, 2019). The hLF1-11 is proposed for intravenous usage in immunocompromised recipients of stem cell transplants for treating both bacterial and fungal infections; novexatin, a cationic peptide generated from defensins, is suggested for treating fungal toe infections; and pexiganan, an analog of peptide magainin (extracted from the skin of *Xenopus laevis*) with 22 amino acid residues, shows robust antimicrobial activity against bacterial and fungal pathogens. In addition, human histatin-based PAC-113 is also under clinical trials as a mouth rinse for oral candidiasis in patients with human immunodeficiency virus (HIV). AFPs undergoing clinical trials are generally designed for topical use because topical administration of peptides can overcome the inherent limitation related to poor stability in physiological fluids, due to their susceptibility to proteases. In addition, in certain areas of the body, such as skin, oral cavity, and vagina, where fungal infections may occur, physiological pH values and salt concentrations are compatible with the optimum activity of AFPs (Duncan and O’Neil, 2013; Wei et al., 2007; Lombardi et al., 2015).

Emerging resistance to conventional antifungals and serious side effects of drugs currently available demand urgent development of novel strategies for protection against fungal pathogens. This goal can be achieved with combination therapy, in which conventional antifungals are used together with other antifungal drugs or AFPs to increase the treatment efficacy compared to single-drug therapy. For instance, lactoferrin-derived peptides Lf (1–11) and bLfcin both exhibit synergy with azole and amphotericin B, which reduce the minimal inhibitory concentrations against *Candida* spp. (Wakabayashi et al., 1996; Lupetti et al., 2003; Fernandes and Carter, 2017). AFPs may also be conjugated with virus-like particles, such as rotavirus VP6 inner capsid protein, to deliver the peptides at the site of infection (Bugli et al., 2014). Likewise, nanoparticles consisting of self-assembled amphiphilic peptides can be generated. Nanotechnology can offer a better delivery system for targeted therapy (Kovalainen et al., 2015). For example, the histatin 5-conjugated polymer-based AmB-delivery carrier system, which is redox-sensitive and pH-responsive, acts both as a synergistic molecule and as a targeting ligand against *C. albicans* (Park et al., 2017).

Fungal growth and consequent mycotoxin release in food and feed pose a serious risk to human health, which may lead to death in acute cases. Therefore, control and prevention of fungal pathogens and foodborne poisoning are among the main tasks of public health we face today. AFPs have been shown to possess both antifungal and anti-mycotoxin biosynthesis activities and thus meet the desired requirements to fight fungal contaminations. Several AFPs have been approved for applications in the food industry as preservatives, such as lactoferrin certified by the European Food Safety Authority (EFSA) since 2012 (EFSA Panel on Dietetic Products, Nutrition and Allergies (NDA), 2012). Mycotoxins commonly found in the food industry include aflatoxins (AFs), deoxynivalenol (DON), ochratoxin A (OTA), zearalenone (ZEA), fumonisins (FUM), patulin (PAT), and citrinin (CIT) (Marin et al., 2013). A few AFPs have been shown to inhibit mycotoxin biosynthesis. For example, peptide cyclo-L-leucyl-L-prolyl from *Achromobacter xylosoxidans* inhibits AF production by suppressing the expression of the AF biosynthesis regulatory gene *aflR* (Yan et al., 2004). Similarly, peptides cyclo-L-Val-L-Pro and cyclo-L-Ala-L-Pro both can inhibit AF biosynthesis of *Aspergillus parasiticus* and *A. flavus* by reducing the mRNA level of *aflR* and blocking the production of norsorolinic acid, a biosynthetic intermediate involved in an early step of AF biosynthetic pathway (Jermnak et al., 2013). In addition, iturin A from *B. subtilis* has been shown to inhibit OTA production by *Aspergillus carbonarius* (Jiang et al., 2020), and AgAFP peptide produced by *Aspergillus giganteus* has been shown to decrease DON production by the genus *Fusarium* (Barakat et al., 2010). Lipopeptides, such as surfactins and fengycins from *Bacillus* species, also have the capacity to inhibit mycotoxin synthesis (Martínez-Culebras et al., 2021).

Given the huge clinical and market needs, the development of novel AFPs and detailed studies on existing AFPs are necessary and urgent. Computer-aided drug design has become an integral part of AFP discovery and development efforts in the pharmaceutical and biotechnology fields. Sequencing has produced a large number of databases including genomic, transcriptomic, proteomic, and functional information. Progresses in the field of molecular biology, analysis of whole genomes, and high-throughput screening of natural and synthetic compounds have resulted in the identification and characterization of new targets, novel scaffolds, and leading structures for potential AFP candidates. Definitely, modern *in silico* molecular modeling techniques will make the screening and discovery of new AFPs more efficient and faster. At the same time, continuing efforts are required to develop and improve natural/modified AFPs, or their analogs/mimics, with high efficiency and a low risk of resistant pathogen emergence. In addition, more efforts should focus on combination therapy, where the synergy between AFPs and conventional antifungal drugs is the main objective. This approach can promote their effectiveness while reducing their toxicity to the host. Last but not least, the modes of action of AFPs still demand further investigation, especially in combination with animal models (Capilla et al., 2007; Hohl, 2014; Van Dijck et al., 2018).

## References

[ref1] Aerts A. M. François I. E. Cammue B. P. Thevissen K. (2008). The mode of antifungal action of plant, insect and human defensins. CMLS 65, 2069–2079. doi: 10.1007/s00018-008-8035-0, PMID: 18360739 PMC11131863

[ref2] Alcouloumre M. S. Ghannoum M. A. Ibrahim A. S. Selsted M. E. Edwards J. E. Jr. (1993). Fungicidal properties of defensin NP-1 and activity against *Cryptococcus neoformans* in vitro. Antimicrob. Agents Chemother. 37, 2628–2632. doi: 10.1128/AAC.37.12.2628, PMID: 8109927 PMC192760

[ref3] Amaral A. C. Silva O. N. Mundim N. C. de Carvalho M. J. Migliolo L. Leite J. R.. (2012). Predicting antimicrobial peptides from eukaryotic genomes: in silico strategies to develop antibiotics. Peptides 37, 301–308. doi: 10.1016/j.peptides.2012.07.021, PMID: 22884922

[ref4] An M. Y. Gao J. Zhao X. F. Wang J. X. (2016). A new subfamily of penaeidin with an additional serine-rich region from kuruma shrimp (*Marsupenaeus japonicus*) contributes to antimicrobial and phagocytic activities. Dev. Comp. Immunol. 59, 186–198. doi: 10.1016/j.dci.2016.02.001, PMID: 26855016

[ref5] Asensio-Calavia P. González-Acosta S. Otazo-Pérez A. López M. R. Morales-delaNuez A. Pérez de la Lastra J. M. (2023). Teleost Piscidins-in silico perspective of natural peptide antibiotics from marine sources. Antibiotics 12:855. doi: 10.3390/antibiotics12050855, PMID: 37237758 PMC10215159

[ref6] Auchtung T. A. Fofanova T. Y. Stewart C. J. Nash A. K. Wong M. C. Gesell J. R.. (2018). Investigating colonization of the healthy adult gastrointestinal tract by fungi. mSphere 3:e00092–18. doi: 10.1128/mSphere.00092-18 29600282 PMC5874442

[ref7] Ayroza G. Ferreira I. L. Sayegh R. S. Tashima A. K. da Silva Junior P. I. (2012). Juruin: an antifungal peptide from the venom of the Amazonian pink toe spider, *Avicularia juruensis*, which contains the inhibitory cystine knot motif. Front. Microbiol. 3:324. doi: 10.3389/fmicb.2012.00324, PMID: 22973266 PMC3437525

[ref8] Baldrian P. Větrovský T. Lepinay C. Kohout P. (2021). High-throughput sequencing view on the magnitude of global fungal diversity. Fungal Divers. 19, 1–9.

[ref9] Barakat H. Spielvogel A. Hassan M. El-Desouky A. El-Mansy H. Rath F.. (2010). The antifungal protein AFP from aspergillus giganteus prevents secondary growth of different fusarium species on barley. Appl. Microbiol. Biotechnol. 87, 617–624. doi: 10.1007/s00253-010-2508-4, PMID: 20217075

[ref10] Barbault F. Landon C. Guenneugues M. Meyer J. P. Schott V. Dimarcq J. L.. (2003). Solution structure of Alo-3: a new knottin-type antifungal peptide from the insect Acrocinus longimanus. Biochemistry 42, 14434–14442. doi: 10.1021/bi035400o, PMID: 14661954

[ref11] Bazinet L. Firdaous L. (2013). Separation of bioactive peptides by membrane processes: technologies and devices. Recent Pat. Biotechnol. 7, 9–27. doi: 10.2174/1872208311307010003, PMID: 23003009

[ref12] Berrocal-Lobo M. Segura A. Moreno M. López G. García-Olmedo F. Molina A. (2002). Snakin-2, an antimicrobial peptide from potato whose gene is locally induced by wounding and responds to pathogen infection. Plant Physiol. 128, 951–961. doi: 10.1104/pp.010685, PMID: 11891250 PMC152207

[ref13] Bondaryk M. Staniszewska M. Zielińska P. Urbańczyk-Lipkowska Z. (2017). Natural antimicrobial peptides as inspiration for design of a new generation antifungal compounds. J. Fungi 3:46. doi: 10.3390/jof3030046, PMID: 29371563 PMC5715947

[ref14] Bormann C. Baier D. Hörr I. Raps C. Berger J. Jung G.. (1999). Characterization of a novel, antifungal, chitin-binding protein from *Streptomyces tendae* Tü901 that interferes with growth polarity. J. Bacteriol. 181, 7421–7429. doi: 10.1128/JB.181.24.7421-7429.1999, PMID: 10601197 PMC94197

[ref15] Boukaew S. Zhang Z. Prasertsan P. Igarashi Y. (2023). Antifungal and antiaflatoxigenic mechanism activity of freeze-dried culture filtrate of Streptomyces philanthi RL-1-178 on the two aflatoxigenic fungi and identification of its active component. J. Appl. Microbiol. 134:lxac091. doi: 10.1093/jambio/lxac091, PMID: 36724264

[ref16] Bowman S. M. Free S. J. (2006). The structure and synthesis of the fungal cell wall. BioEssays 28, 799–808. doi: 10.1002/bies.20441 16927300

[ref17] Brady R. Woonton B. Gee M. L. O’Connor A. J. (2008). Hierarchical mesoporous silica materials for separation of functional food ingredients — a review. Innov. Food Sci. Emerg. Technol. 9, 243–248. doi: 10.1016/j.ifset.2007.10.002

[ref18] Brown G. D. Denning D. W. Gow N. A. Levitz S. M. Netea M. G. White T. C. (2012). Hidden killers: human fungal infections. Sci. Transl. Med. 4:165rv13. doi: 10.1126/scitranslmed.3004404, PMID: 23253612

[ref19] Bugli F. Caprettini V. Cacaci M. Martini C. Paroni Sterbini F. Torelli R.. (2014). Synthesis and characterization of different immunogenic viral nanoconstructs from rotavirus VP6 inner capsid protein. Int. J. Nanomedicine 9, 2727–2739. doi: 10.2147/IJN.S60014, PMID: 24936129 PMC4047981

[ref20] Cabib E. Arroyo J. (2013). How carbohydrates sculpt cells: chemical control of morphogenesis in the yeast cell wall. Nat. Rev. Microbiol. 11, 648–655. doi: 10.1038/nrmicro3090, PMID: 23949603

[ref21] Campbell-Platt G. Cook P. E. (2008). Fungi in the production of foods and food ingredients. J. Appl. Microbiol. 67, 117s–131s. doi: 10.1111/j.1365-2672.1989.tb03776.x

[ref22] Capilla J. Clemons K. V. Stevens D. A. (2007). Animal models: an important tool in mycology. Med. Mycol. 45, 657–684. doi: 10.1080/13693780701644140, PMID: 18027253 PMC7107685

[ref23] Chahardoli M. Fazeli A. Ghabooli M. (2018). Recombinant production of bovine lactoferrin-derived antimicrobial peptide in tobacco hairy roots expression system. Plant Physiol. Biochem. 123, 414–421. doi: 10.1016/j.plaphy.2017.12.037, PMID: 29310078

[ref24] Cheng R. Xu Q. Hu F. Li H. Yang B. Duan Z.. (2021). Antifungal activity of MAF-1A peptide against *Candida albicans*. Int. Microbiol. Off. J. Spanish Soc. Microbiol. 24, 233–242. doi: 10.1007/s10123-021-00159-z, PMID: 33452940 PMC8046747

[ref25] Chiu T. Poucet T. Li Y. (2022). The potential of plant proteins as antifungal agents for agricultural applications. Synth. Syst. Biotechnol. 7, 1075–1083. doi: 10.1016/j.synbio.2022.06.009, PMID: 35891944 PMC9305310

[ref26] Choi H. Hwang J. S. Lee D. G. (2013). Antifungal effect and pore-forming action of lactoferricin B like peptide derived from centipede *Scolopendra subspinipes* mutilans. Biochim. Biophys. Acta 1828, 2745–2750. doi: 10.1016/j.bbamem.2013.07.021, PMID: 23896552

[ref27] Ciociola T. Giovati L. Conti S. Magliani W. Santinoli C. Polonelli L. (2016). Natural and synthetic peptides with antifungal activity. Future Med. Chem. 8, 1413–1433. doi: 10.4155/fmc-2016-0035 27502155

[ref28] Cole A. M. Weis P. Diamond G. (1997). Isolation and characterization of pleurocidin, an antimicrobial peptide in the skin secretions of winter flounder. J. Biol. Chem. 272, 12008–12013. doi: 10.1074/jbc.272.18.12008, PMID: 9115266

[ref29] Cuthbertson B. J. Shepard E. F. Chapman R. W. Gross P. S. (2002). Diversity of the penaeidin antimicrobial peptides in two shrimp species. Immunogenetics 54, 442–445. doi: 10.1007/s00251-002-0487-z 12242595

[ref30] Dash R. Bhattacharjya S. (2021). Thanatin: An emerging host defense antimicrobial peptide with multiple modes of action. Int. J. Mol. Sci. 22:1522. doi: 10.3390/ijms22041522, PMID: 33546369 PMC7913509

[ref31] D'Auria F. D. Casciaro B. De Angelis M. Marcocci M. E. Palamara A. T. Nencioni L.. (2022). Antifungal activity of the frog skin peptide Temporin G and its effect on *Candida albicans* virulence factors. Int. J. Mol. Sci. 23:6345. doi: 10.3390/ijms23116345, PMID: 35683025 PMC9181532

[ref32] De Lucca A. J. (2000). Antifungal peptides: potential candidates for the treatment of fungal infections. Expert Opin. Investig. Drugs 9, 273–299. doi: 10.1517/13543784.9.2.273 11060677

[ref33] De Lucca A. J. Walsh T. J. (1999). Antifungal peptides: novel therapeutic compounds against emerging pathogens. Antimicrob. Agents Chemother. 43, 1–11. doi: 10.1128/AAC.43.1.1, PMID: 9869556 PMC89011

[ref34] Destoumieux D. Munoz M. Bulet P. Bachère E. (2000). Penaeidins, a family of antimicrobial peptides from penaeid shrimp (Crustacea, Decapoda). Cell. Mol. Life Sci. 57, 1260–1271. doi: 10.1007/pl00000764 11028917 PMC11146768

[ref35] Drago-Serrano M. E. Campos-Rodriguez R. Carrero J. C. de la Garza M. (2018). Lactoferrin and peptide-derivatives: antimicrobial agents with potential use in nonspecific immunity modulation. Curr. Pharm. Des. 24, 1067–1078. doi: 10.2174/1381612824666180327155929, PMID: 29589540

[ref36] Dubos R. J. (1939). Studies on a bactericidal agent extracted from a soil bacillus: II. Protective effect of the bactericidal agent against experimental pneumococcus infections in mice. J. Exp. Med. 70, 11–17. doi: 10.1084/jem.70.1.11, PMID: 19870886 PMC2133780

[ref37] Duncan V. M. S. O’Neil D. A. (2013). Commercialization of antifungal peptides. Fungal Biol. Rev. 26, 156–165. doi: 10.1016/j.fbr.2012.11.001

[ref38] EFSA Panel on Dietetic Products, Nutrition and Allergies (NDA) (2012). Scientific opinion on bovine lactoferrin. EFSA J. 10:2811. doi: 10.2903/j.efsa.2012.2811 PMC13093088 42016111

[ref39] Emri T. Majoros L. Tóth V. Pócsi I. (2013). Echinocandins: production and applications. Appl. Microbiol. Biotechnol. 97, 3267–3284. doi: 10.1007/s00253-013-4761-9 23463246

[ref40] Endo M. Takesako K. Kato I. Yamaguchi H. (1997). Fungicidal action of aureobasidin a, a cyclic depsipeptide antifungal antibiotic, against *Saccharomyces cerevisiae*. Antimicrob. Agents Chemother. 41, 672–676. doi: 10.1128/AAC.41.3.672, PMID: 9056012 PMC163770

[ref41] Faruck M. O. Yusof F. Chowdhury S. (2016). An overview of antifungal peptides derived from insect. Peptides 80, 80–88. doi: 10.1016/j.peptides.2015.06.001, PMID: 26093218

[ref42] Fehlbaum P. Bulet P. Chernysh S. Briand J. P. Roussel J. P. Letellier L.. (1996). Structure-activity analysis of thanatin, a 21-residue inducible insect defense peptide with sequence homology to frog skin antimicrobial peptides. Proc. Natl. Acad. Sci. USA 93, 1221–1225. doi: 10.1073/pnas.93.3.1221, PMID: 8577744 PMC40060

[ref43] Fehlbaum P. Bulet P. Michaut L. Lagueux M. Broekaert W. F. Hetru C.. (1994). Insect immunity. Septic injury of Drosophila induces the synthesis of a potent antifungal peptide with sequence homology to plant antifungal peptides. J. Biol. Chem. 269, 33159–33163. doi: 10.1016/S0021-9258(20)30111-3, PMID: 7806546

[ref44] Fennell J. F. Shipman W. H. Cole L. J. (1967). Antibacterial action of a bee venom fraction (melittin) against a penicillin-resistant staphylococcus and other microorganisms. USNRDL-TR-67-101. Res. Dev. Tech. Rep., 1–13. doi: 10.21236/ad0658324, PMID: 5300771

[ref45] Fernandes K. E. Carter D. A. (2017). The antifungal activity of lactoferrin and its derived peptides: mechanisms of action and synergy with drugs against fungal pathogens. Front. Microbiol. 8:2. doi: 10.3389/fmicb.2017.00002, PMID: 28149293 PMC5241296

[ref46] Fernandes K. E. Weeks K. Carter D. A. (2020). Lactoferrin is broadly active against yeasts and highly synergistic with amphotericin B. Antimicrob. Agents Chemother. 64, e02284–e02219. doi: 10.1128/AAC.02284-19, PMID: 32094132 PMC7179636

[ref47] Fernández de Ullivarri M. Arbulu S. Garcia-Gutierrez E. Cotter P. D. (2020). Antifungal peptides as therapeutic agents. Front. Cell. Infect. Microbiol. 10:105. doi: 10.3389/fcimb.2020.00105, PMID: 32257965 PMC7089922

[ref48] Fisher M. C. Hawkins N. J. Sanglard D. Gurr S. J. (2018). Worldwide emergence of resistance to antifungal drugs challenges human health and food security. Science 360, 739–742. doi: 10.1126/science.aap7999 29773744

[ref49] Fleet G. H. (1991). “Cell walls” in The yeasts. eds. Rose A. H. Harrison J. S.. 2nd ed (New York, NY: Academic Press), 199–277.

[ref50] Gamaletsou M. N. Walsh T. J. Sipsas N. V. (2018). Invasive fungal infections in patients with hematological malignancies: emergence of resistant pathogens and new antifungal therapies. Turk. J. Haematol. 35, 1–11. doi: 10.4274/tjh.2018.0007, PMID: 29391334 PMC5843768

[ref51] Games P. D. Dos Santos I. S. Mello E. O. Diz M. S. Carvalho A. O. de Souza-Filho G. A. (2008). Isolation, characterization and cloning of a cDNA encoding a new antifungal defensin from *Phaseolus vulgaris* L. seeds. Peptides 29, 2090–2100. doi: 10.1016/j.peptides.2008.08.008, PMID: 18786582

[ref52] Gao A.-G. Hakimi S. M. Mittanck C. A. Wu Y. Woerner B. M. Stark D. M.. (2000). Fungal pathogen protection in potato by expression of a plant defensin peptide. Nat. Biotechnol. 18, 1307–1310. doi: 10.1038/82436, PMID: 11101813

[ref53] Gennaro R. Zanetti M. (2000). Structural features and biological activities of the cathelicidin-derived antimicrobial peptides. Biopolymers 55, 31–49. doi: 10.1002/1097-0282(2000)55:1<31::AID-BIP40>3.0.CO;2-9 10931440

[ref54] Gong Y. Li H. Wu F. Li Y. Zhang S. (2022). Fungicidal activity of AP10W, a short peptide derived from AP-2 complex subunit mu-a, in vitro and in vivo. Biomolecules 12:965. doi: 10.3390/biom12070965, PMID: 35883521 PMC9313395

[ref55] Gupta A. Gupta R. Singh R. L. (2017). Microbes and environment. Principles and applications of environmental biotechnology for a sustainable future. Cham: Springer, 43–84.

[ref56] Habermann E. (1972). Bee and wasp venoms. Science 177, 314–322. doi: 10.1126/science.177.4046.314 4113805

[ref57] Hagen S. Marx F. Ram A. F. Meyer V. (2007). The antifungal protein AFP from aspergillus giganteus inhibits chitin synthesis in sensitive fungi. Appl. Environ. Microbiol. 73, 2128–2134. doi: 10.1128/AEM.02497-06, PMID: 17277210 PMC1855660

[ref58] Hancock R. E. (2000). Cationic antimicrobial peptides: towards clinical applications. Expert Opin. Investig. Drugs 9, 1723–1729. doi: 10.1517/13543784.9.8.1723, PMID: 11060771

[ref59] Hancock R. E. Chapple D. S. (1999). Peptide antibiotics. Antimicrob. Agents Chemother. 43, 1317–1323. doi: 10.1128/AAC.43.6.1317, PMID: 10348745 PMC89271

[ref60] Haney E. F. Straus S. K. Hancock R. E. W. (2019). Reassessing the host defense peptide landscape. Front. Chem. 7:43. doi: 10.3389/fchem.2019.00043, PMID: 30778385 PMC6369191

[ref61] Helmerhorst E. J. Troxler R. F. Oppenheim F. G. (2001). The human salivary peptide histatin 5 exerts its antifungal activity through the formation of reactive oxygen species. Proc. Natl. Acad. Sci. USA 98, 14637–14642. doi: 10.1073/pnas.141366998, PMID: 11717389 PMC64734

[ref62] Hohl T. M. (2014). Overview of vertebrate animal models of fungal infection. J. Immunol. Methods 410, 100–112. doi: 10.1016/j.jim.2014.03.022, PMID: 24709390 PMC4163114

[ref63] Holaskova E. Galuszka P. Frebort I. Oz M. T. (2015). Antimicrobial peptide production and plant-based expression systems for medical and agricultural biotechnology. Biotechnol. Adv. 33, 1005–1023. doi: 10.1016/j.biotechadv.2015.03.007, PMID: 25784148

[ref64] Hollmann A. Martinez M. Maturana P. Semorile L. C. Maffia P. C. (2018). Antimicrobial peptides: interaction with model and biological membranes and synergism with chemical antibiotics. Front. Chem. 6:204. doi: 10.3389/fchem.2018.00204 29922648 PMC5996110

[ref65] Huber A. Hajdu D. Bratschun-Khan D. Gáspári Z. Varbanov M. Philippot S.. (2018). New antimicrobial potential and structural properties of PAFB: a cationic, cysteine-rich protein from Penicillium chrysogenum Q176. Sci. Rep. 8:1751. doi: 10.1038/s41598-018-20002-2, PMID: 29379111 PMC5788923

[ref66] Huffnagle G. B. Noverr M. C. (2013). The emerging world of the fungal microbiome. Trends Microbiol. 21, 334–341. doi: 10.1016/j.tim.2013.04.002, PMID: 23685069 PMC3708484

[ref67] Hwang J. S. Lee J. Hwang B. Nam S. H. Yun E. Y. Kim S. R.. (2010). Isolation and characterization of Psacotheasin, a novel Knottin-type antimicrobial peptide, from Psacothea hilaris. J. Microbiol. Biotechnol. 20, 708–711. doi: 10.4014/jmb.1002.02003, PMID: 20467242

[ref68] Hwang J. S. Lee J. Kim Y. J. Bang H. S. Yun E. Y. Kim S. R.. (2009). Isolation and characterization of a Defensin-like peptide (Coprisin) from the dung beetle, *Copris tripartitus*. Int. J. Pept. 2009:136284. doi: 10.1155/2009/136284, PMID: 20721297 PMC2915626

[ref69] Jermnak U. Chinaphuti A. Poapolathep A. Kawai R. Nagasawa H. Sakuda S. (2013). Prevention of aflatoxin contamination by a soil bacterium of *Stenotrophomonas* sp. that produces aflatoxin production inhibitors. Microbiology 159, 902–912. doi: 10.1099/mic.0.065813-0, PMID: 23449921

[ref70] Jiang C. Li Z. Shi Y. Guo D. Pang B. Chen X.. (2020). *Bacillus subtilis* inhibits aspergillus Carbonarius by producing Iturin a, which disturbs the transport, energy metabolism, and osmotic pressure of fungal cells as revealed by transcriptomics analysis. Int. J. Food Microbiol. 330:108783. doi: 10.1016/j.ijfoodmicro.2020.108783, PMID: 32659523

[ref71] Joly S. Maze C. McCray P. B. Jr. Guthmiller J. M. (2004). Human beta-defensins 2 and 3 demonstrate strain-selective activity against oral microorganisms. J. Clin. Microbiol. 42, 1024–1029. doi: 10.1128/JCM.42.3.1024-1029.2004, PMID: 15004048 PMC356847

[ref72] Kaiserer L. Oberparleiter C. Weiler-Görz R. Burgstaller W. Leiter E. Marx F. (2003). Characterization of the Penicillium chrysogenum antifungal protein PAF. Arch. Microbiol. 180, 204–210. doi: 10.1007/s00203-003-0578-8, PMID: 12856109

[ref73] Kang Y. Carlson R. Tharpe W. Schell M. A. (1998). Characterization of genes involved in biosynthesis of a novel antibiotic from *Burkholderia cepacia* BC11 and their role in biological control of Rhizoctonia solani. Appl. Environ. Microbiol. 64, 3939–3947. doi: 10.1128/AEM.64.10.3939-3947.1998, PMID: 9758823 PMC106582

[ref74] Kang H. K. Seo C. H. Park Y. (2015). Marine peptides and their anti-infective activities. Mar. Drugs 13, 618–654. doi: 10.3390/md13010618, PMID: 25603351 PMC4306955

[ref75] Kapitan M. Niemiec M. J. Steimle A. Frick J. S. Jacobsen I. D. (2018). Fungi as part of the microbiota and interactions with intestinal bacteria. Curr. Top. Microbiol. Immunol. 422, 265–301. doi: 10.1007/82_2018_117 30062595

[ref76] Karasuda S. Tanaka S. Kajihara H. Yamamoto Y. Koga D. (2003). Plant chitinase as a possible biocontrol agent for use instead of chemical fungicides. Biosci. Biotechnol. Biochem. 67, 221–224. doi: 10.1271/bbb.67.221, PMID: 12619703

[ref77] Kathiravan M. K. Salake A. B. Chothe A. S. Dudhe P. B. Watode R. P. Mukta M. S.. (2012). The biology and chemistry of antifungal agents: a review. Bioorg. Med. Chem. 20, 5678–5698. doi: 10.1016/j.bmc.2012.04.045 22902032

[ref78] Kavanagh K. Dowd S. (2004). Histatins: antimicrobial peptides with therapeutic potential. J. Pharm. Pharmacol. 56, 285–289. doi: 10.1211/0022357022971 15025852

[ref79] Kim S. R. Hong M. Y. Park S. W. Choi K. H. Yun E. Y. Goo T. W.. (2010). Characterization and cDNA cloning of a cecropin-like antimicrobial peptide, papiliocin, from the swallowtail butterfly, *Papilio xuthus*. Mol. Cells. 29, 419–424. doi: 10.1007/s10059-010-0050-y, PMID: 20213310

[ref80] Kim S. Hwang J. S. Lee D. G. (2020). Lactoferricin B like peptide triggers mitochondrial disruption-mediated apoptosis by inhibiting respiration under nitric oxide accumulation in *Candida albicans*. IUBMB Life 72, 1515–1527. doi: 10.1002/iub.2284 32267619

[ref81] Klich M. A. Lax A. R. Bland J. M. (1991). Inhibition of some mycotoxigenic fungi by iturin a, a peptidolipid produced by *Bacillus subtilis*. Mycopathologia 116, 77–80. doi: 10.1007/BF00436368, PMID: 1780001

[ref82] Koo H. B. Seo J. (2019). Antimicrobial peptides under clinical investigation. J. Pept. Sci. 111:e24122. doi: 10.1002/pep2.24122

[ref83] Kovalainen M. Monkare J. Riikonen J.. (2015). Novel delivery systems for improving the clinical use of peptides. Pharmacol. Rev. 67, 541–561. doi: 10.1124/pr.113.008367 26023145

[ref84] Lamberty M. Caille A. Landon C. Tassin-Moindrot S. Hetru C. Bulet P.. (2001a). Solution structures of the antifungal heliomicin and a selected variant with both antibacterial and antifungal activities. Biochemistry 40, 11995–12003. doi: 10.1021/bi0103563, PMID: 11580275

[ref85] Lamberty M. Zachary D. Lanot R. Bordereau C. Robert A. Hoffmann J. A.. (2001b). Insect immunity. Constitutive expression of a cysteine-rich antifungal and a linear antibacterial peptide in a termite insect. J. Biol. Chem. 276, 4085–4092. doi: 10.1074/jbc.M002998200 11053427

[ref86] Landon C. Barbault F. Legrain M. Menin L. Guenneugues M. Schott V.. (2004). Lead optimization of antifungal peptides with 3D NMR structures analysis. Protein Sci. 13, 703–713. doi: 10.1110/ps.03404404 14978308 PMC2286723

[ref87] Le C. F. Fang C. M. Sekaran S. D. (2017). Intracellular targeting mechanisms by antimicrobial peptides. Antimicrob. Agents Chemother. 61, e02340–e02316. doi: 10.1128/AAC.02340-16, PMID: 28167546 PMC5365711

[ref88] Lee J. Hwang J. S. Hwang I. S. Cho J. Lee E. Kim Y.. (2012). Coprisin-induced antifungal effects in *Candida albicans* correlate with apoptotic mechanisms. Free Radic. Biol. Med. 52, 2302–2311. doi: 10.1016/j.freeradbiomed.2012.03.012, PMID: 22542795

[ref89] Lee D. G. Kim H. K. Kim S. A. Park Y. Park S. C. Jang S. H.. (2003). Fungicidal effect of indolicidin and its interaction with phospholipid membranes. Biochem. Biophys. Res. Commun. 305, 305–310. doi: 10.1016/s0006-291x(03)00755-1, PMID: 12745074

[ref90] Lehrer R. I. Ganz T. Szklarek D. Selsted M. E. (1988). Modulation of the in vitro candidacidal activity of human neutrophil defensins by target cell metabolism and divalent cations. J. Clin. Invest. 81, 1829–1835. doi: 10.1172/JCI113527, PMID: 3290255 PMC442632

[ref91] Lenardon M. D. Munro C. A. Gow N. A. (2010). Chitin synthesis and fungal pathogenesis. Curr. Opin. Microbiol. 13, 416–423. doi: 10.1016/j.mib.2010.05.002, PMID: 20561815 PMC2923753

[ref92] Lestrade P. P. A. Meis J. F. Melchers W. J. G. Verweij P. E. (2019). Triazole resistance in *Aspergillus fumigatus*: recent insights and challenges for patient management. Clin. Microbiol. Infect. 25, 799–806. doi: 10.1016/j.cmi.2018.11.027, PMID: 30580035

[ref93] Li Y. (2011). Recombinant production of antimicrobial peptides in *Escherichia coli*: a review. Protein Expr. Purif. 80, 260–267. doi: 10.1016/j.pep.2011.08.001, PMID: 21843642

[ref94] Li R. Chen C. Zhang B. Jing H. Wang Z. Wu C.. (2019). The chromogranin A-derived antifungal peptide CGA-N9 induces apoptosis in *Candida tropicalis*. Biochem. J. 476, 3069–3080. doi: 10.1042/BCJ20190483, PMID: 31652303 PMC6824672

[ref95] Liu Z. Yuan K. Zhang R. Ren X. Liu X. Zhao S.. (2016). Cloning and purification of the first termicin-like peptide from the cockroach Eupolyphaga sinensis. J. Venom Anim. Toxins Incl. Trop Dis. 22:5. doi: 10.1186/s40409-016-0058-7, PMID: 26823660 PMC4730610

[ref96] Lobo D. S. Pereira I. B. Fragel-Madeira L. Medeiros L. N. Cabral L. M. Faria J.. (2007). Antifungal *Pisum sativum* defensin 1 interacts with Neurospora crassa cyclin F related to the cell cycle. Biochemistry 46, 987–996. doi: 10.1021/bi061441j, PMID: 17240982

[ref97] Lohner K. Prenner E. J. (1999). Differential scanning calorimetry and X-ray diffraction studies of the specificity of the interaction of antimicrobial peptides with membrane-mimetic systems. Biochim. Biophys. Acta 1462, 141–156. doi: 10.1016/s0005-2736(99)00204-7, PMID: 10590306

[ref98] Lombardi L. Maisetta G. Batoni G. Tavanti A. (2015). Insights into the antimicrobial properties of hepcidins: advantages and drawbacks as potential therapeutic agents. Molecules 20, 6319–6341. doi: 10.3390/molecules20046319 25867823 PMC6272296

[ref99] López-Abarrategui C. Alba A. Silva O. N. Reyes-Acosta O. Vasconcelos I. M. Oliveira J. T. A.. (2012). Functional characterization of a synthetic hydrophilic antifungal peptide derived from the marine snail *Cenchritis muricatus*. Biochimie 94, 968–974. doi: 10.1016/j.biochi.2011.12.016, PMID: 22210491

[ref100] López-Meza J. Ochoa-Zarzosa A. Aguilar J. Loeza-Lara P. (2011). “Antimicrobial peptides: diversity and perspectives for their biomedical application,” in *Biomedical Engineering, Trends, Research and Technologies.* London: IntechOpen.

[ref101] Lücking R. Aime M. C. Robbertse B. Miller A. N. Aoki T. Ariyawansa H. A.. (2021). Fungal taxonomy and sequence-based nomenclature. Nat. Microbiol. 6, 540–548. doi: 10.1038/s41564-021-00888-x, PMID: 33903746 PMC10116568

[ref102] Lupetti A. Paulusma-Annema A. Welling M. M. Dogterom-Ballering H. Brouwer C. P. J. M. Senesi S.. (2003). Synergistic activity of the N-terminal peptide of human lactoferrin and fluconazole against Candida species. Antimicrob. Agents Chemother. 47, 262–267. doi: 10.1128/aac.47.1.262-267.2003, PMID: 12499200 PMC149030

[ref103] Marcocci M. E. Amatore D. Villa S. Casciaro B. Aimola P. Franci G.. (2018). The amphibian antimicrobial peptide Temporin B inhibits in vitro herpes simplex virus 1 infection. Antimicrob. Agents Chemother. 62, e02367–e02317. doi: 10.1128/AAC.02367-17, PMID: 29483113 PMC5923125

[ref104] Marin S. Ramos A. J. Cano-Sancho G. Sanchis V. (2013). Mycotoxins: occurrence, toxicology, and exposure assessment. Food Chem. Toxicol. 60, 218–237. doi: 10.1016/j.fct.2013.07.047 23907020

[ref105] Martínez-Culebras P. V. Gandía M. Garrigues S. Marcos J. F. Manzanares P. (2021). Antifungal peptides and proteins to control toxigenic Fungi and mycotoxin biosynthesis. Int. J. Mol. Sci. 22:13261. doi: 10.3390/ijms222413261, PMID: 34948059 PMC8703302

[ref106] Matejuk A. Leng Q. Begum M. D. Woodle M. C. Scaria P. Chou S. T.. (2010). Peptide-based antifungal therapies against emerging infections. Drugs Future 35:197. doi: 10.1358/dof.2010.35.3.1452077, PMID: 20495663 PMC2873032

[ref107] McCarthy P. J. Troke P. F. Gull K. (1985). Mechanism of action of nikkomycin and the peptide transport system of *Candida albicans*. J. Gen. Microbiol. 131, 775–780. doi: 10.1099/00221287-131-4-775, PMID: 3886837

[ref108] Mello E. O. Ribeiro S. F. Carvalho A. O. Santos I. S. Da Cunha M. Santa-Catarina C.. (2011). Antifungal activity of PvD1 defensin involves plasma membrane permeabilization, inhibition of medium acidification, and induction of ROS in fungi cells. Curr. Microbiol. 62, 1209–1217. doi: 10.1007/s00284-010-9847-3, PMID: 21170711

[ref109] Memariani H. Memariani M. (2020). Anti-fungal properties and mechanisms of melittin. Appl. Microbiol. Biotechnol. 104, 6513–6526. doi: 10.1007/s00253-020-10701-0, PMID: 32500268

[ref110] Memariani M. Memariani H. (2023). Antifungal properties of cathelicidin LL-37: current knowledge and future research directions. World J. Microbiol. Biotechnol. 40:34. doi: 10.1007/s11274-023-03852-5, PMID: 38057654

[ref111] Mendes M. A. de Souza B. M. Marques M. R. Palma M. S. (2004). Structural and biological characterization of two novel peptides from the venom of the neotropical social wasp Agelaia pallipes pallipes. Toxicon 44, 67–74. doi: 10.1016/j.toxicon.2004.04.009, PMID: 15225564

[ref112] Miyata T. Tokunaga F. Yoneya T. Yoshikawa K. Iwanaga S. Niwa M.. (1989). Antimicrobial peptides, isolated from horseshoe crab hemocytes, tachyplesin II, and polyphemusins I and II: chemical structures and biological activity. J. Biochem. 106, 663–668. doi: 10.1093/oxfordjournals.jbchem.a122913, PMID: 2514185

[ref113] Money N. P. (2016). Fungi and biotechnology. J. Fungi 2016, 401–424. doi: 10.1016/B978-0-12-382034-1.00012-8

[ref114] Monincová L. Slaninová J. Fučík V. Hovorka O. Voburka Z. Bednárová L.. (2012). Lasiocepsin, a novel cyclic antimicrobial peptide from the venom of eusocial bee *Lasioglossum laticeps* (Hymenoptera: Halictidae). Amino Acids 43, 751–761. doi: 10.1007/s00726-011-1125-6, PMID: 22038181

[ref115] Moreno-Expósito L. Illescas-Montes R. Melguizo-Rodríguez L. Ruiz C. Ramos-Torrecillas J. de Luna-Bertos E. (2018). Multifunctional capacity and therapeutic potential of lactoferrin. Life Sci. 195, 61–64. doi: 10.1016/j.lfs.2018.01.002, PMID: 29307524

[ref116] Muhialdin B. J. Algboory H. L. Kadum H. Mohammed N. K. Saari N. Hassan Z.. (2020). Antifungal activity determination for the peptides generated by *Lactobacillus plantarum* TE10 against aspergillus flavus in maize seeds. Food Control 109:106898. doi: 10.1016/j.foodcont.2019.106898

[ref117] Mukherjee D. Singh S. Kumar M. Kumar V. Datta S. Dhanjal D. S. (2018). “Fungal biotechnology: role and aspects” in Fungi and their role in sustainable development: Current perspectives. eds. Gehlot P. Singh J. (Singapore: Springer Singapore), 91–103.

[ref118] Nagiec M. M. Nagiec E. E. Baltisberger J. A. Wells G. B. Lester R. L. Dickson R. C. (1997). Sphingolipid synthesis as a target for antifungal drugs. Complementation of the inositol phosphorylceramide synthase defect in a mutant strain of *Saccharomyces cerevisiae* by the AUR1 gene. J. Biol. Chem. 272, 9809–9817. doi: 10.1074/jbc.272.15.9809 9092515

[ref119] Nawrocki K. L. Crispell E. K. McBride S. M. (2014). Antimicrobial peptide resistance mechanisms of gram-positive Bacteria. Antibiotics 3, 461–492. doi: 10.3390/antibiotics3040461, PMID: 25419466 PMC4239024

[ref120] Nolde S. B. Vassilevski A. A. Rogozhin E. A. Barinov N. A. Balashova T. A. Samsonova O. V.. (2011). Disulfide-stabilized helical hairpin structure and activity of a novel antifungal peptide EcAMP1 from seeds of barnyard grass (*Echinochloa crus-galli*). J. Biol. Chem. 286, 25145–25153. doi: 10.1074/jbc.M110.200378, PMID: 21561864 PMC3137087

[ref121] Notomista E. Falanga A. Fusco S. Pirone L. Zanfardino A. Galdiero S.. (2015). The identification of a novel Sulfolobus islandicus CAMP-like peptide points to archaeal microorganisms as cell factories for the production of antimicrobial molecules. Microb. Cell Factories 14:126. doi: 10.1186/s12934-015-0302-9, PMID: 26338197 PMC4559164

[ref122] Odintsova T. I. Vassilevski A. A. Slavokhotova A. A. Musolyamov A. K. Finkina E. I. Khadeeva N. V.. (2009). A novel antifungal hevein-type peptide from Triticum kiharae seeds with a unique 10-cysteine motif. FEBS J. 276, 4266–4275. doi: 10.1111/j.1742-4658.2009.07135.x, PMID: 19583772

[ref123] Oppenheim F. G. Xu T. McMillian F. M. Levitz S. M. Diamond R. D. Offner G. D.. (1988). Histatins, a novel family of histidine-rich proteins in human parotid secretion. Isolation, characterization, primary structure, and fungistatic effects on *Candida albicans*. J. Biol. Chem. 263, 7472–7477. doi: 10.1016/S0021-9258(18)68522-9, PMID: 3286634

[ref124] Osaki T. Omotezako M. Nagayama R. Hirata M. Iwanaga S. Kasahara J.. (1999). Horseshoe crab hemocyte-derived antimicrobial polypeptides, tachystatins, with sequence similarity to spider neurotoxins. J. Biol. Chem. 274, 26172–26178. doi: 10.1074/jbc.274.37.26172, PMID: 10473569

[ref125] Osborn R. W. de Samblanx G. W. Thevissen K. Goderis I. Torrekens S. van Leuven F.. (1995). Isolation and characterisation of plant defensins from seeds of Asteraceae, Fabaceae, Hippocastanaceae and Saxifragaceae. FEBS Lett. 368, 257–262. doi: 10.1016/0014-5793(95)00666-w, PMID: 7628617

[ref126] Ovchinnikova T. V. Aleshina G. M. Balandin S. V. Krasnosdembskaya A. D. Markelov M. L. Frolova E. I.. (2004). Purification and primary structure of two isoforms of arenicin, a novel antimicrobial peptide from marine polychaeta *Arenicola marina*. FEBS Lett. 577, 209–214. doi: 10.1016/j.febslet.2004.10.012 15527787

[ref127] Padovan L. Segat L. Pontillo A. Antcheva N. Tossi A. Crovella S. (2010). Histatins in non-human primates: gene variations and functional effects. Protein Pept. Lett. 17, 909–918. doi: 10.2174/092986610791306715 20423320

[ref128] Park S.-C. Kim Y.-M. Lee J.-K. Kim N.-H. Kim E.-J. Heo H.. (2017). Targeting and synergistic action of an antifungal peptide in an antibiotic drug-delivery system. J. Control. Release 256, 46–55. doi: 10.1016/j.jconrel.2017.04.023, PMID: 28428067

[ref129] Park C. B. Lee J. H. Park I. Y. Kim M. S. Kim S. C. (1997). A novel antimicrobial peptide from the loach, *Misgurnus anguillicaudatus*. FEBS Lett. 411, 173–178. doi: 10.1016/s0014-5793(97)00684-4, PMID: 9271200

[ref130] Pei J. Feng Z. Ren T. Sun H. Han H. Jin W.. (2018). Purification, characterization and application of a novel antimicrobial peptide from *Andrias davidianus* blood. Lett. Appl. Microbiol. 66, 38–43. doi: 10.1111/lam.12823 29130500

[ref131] Pesic A. Baumann H. I. Kleinschmidt K. Ensle P. Wiese J. Süssmuth R. D.. (2013). Champacyclin, a new cyclic octapeptide from Streptomyces strain C42 isolated from the Baltic Sea. Mar. Drugs 11, 4834–4857. doi: 10.3390/md11124834, PMID: 24317473 PMC3877890

[ref132] Pfaller M. A. Huband M. D. Flamm R. K. Bien P. A. Castanheira M. (2019). In vitro activity of APX001A (Manogepix) and comparator agents against 1,706 fungal isolates collected during an international surveillance program in 2017. Antimicrob. Agents Chemother. 63, e00840–e00819. doi: 10.1128/AAC.00840-19, PMID: 31182527 PMC6658749

[ref133] Phukhamsakda C. Nilsson R. H. Bhunjun C. S. de Farias A. R. G. Sun Y. R. Wijesinghe S. N.. (2022). The numbers of fungi: contributions from traditional taxonomic studies and challenges of metabarcoding. Fungal Divers. 114, 327–386. doi: 10.1007/s13225-022-00502-3

[ref134] Pólvora T. L. S. Nobre Á. V. V. Tirapelli C. Taba M. Jr. Macedo L. D. Santana R. C.. (2018). Relationship between human immunodeficiency virus (HIV-1) infection and chronic periodontitis. Expert Rev. Clin. Immunol. 14, 315–327. doi: 10.1080/1744666X.2018.1459571, PMID: 29595347

[ref135] Porto W. F. Silva O. N. Franco O. L. (2012). “Prediction and rational design of antimicrobial peptides,” in *Protein Structure Eshel Faraggi* (New York, NY: IntechOpen).

[ref136] Rakers S. Niklasson L. Steinhagen D. Kruse C. Schauber J. Sundell K.. (2013). Antimicrobial peptides (AMPs) from fish epidermis: perspectives for investigative dermatology. J. Invest. Dermatol. 133, 1140–1149. doi: 10.1038/jid.2012.503, PMID: 23407389

[ref137] Ramamoorthy V. Zhao X. Snyder A. K. Xu J. R. Shah D. M. (2007). Two mitogen-activated protein kinase signalling cascades mediate basal resistance to antifungal plant defensins in fusarium graminearum. Cell. Microbiol. 9, 1491–1506. doi: 10.1111/j.1462-5822.2006.00887.x, PMID: 17253976

[ref138] Rascón-Cruz Q. Espinoza-Sánchez E. A. Siqueiros-Cendón T. S. Nakamura-Bencomo S. I. Arévalo-Gallegos S. Iglesias-Figueroa B. F. (2021). Lactoferrin: a glycoprotein involved in immunomodulation, anticancer, and antimicrobial processes. Molecules 26:205. doi: 10.3390/molecules26010205, PMID: 33401580 PMC7795860

[ref139] Rodríguez-Martín A. Acosta R. Liddell S. Núñez F. Benito M. J. Asensio M. A. (2010). Characterization of the novel antifungal protein PgAFP and the encoding gene of Penicillium chrysogenum. Peptides 31, 541–547. doi: 10.1016/j.peptides.2009.11.002, PMID: 19914321

[ref140] Rogozhin E. A. Slezina M. P. Slavokhotova A. A. Istomina E. A. Korostyleva T. V. Smirnov A. N.. (2015). A novel antifungal peptide from leaves of the weed *Stellaria media* L. Biochimie 116, 125–132. doi: 10.1016/j.biochi.2015.07.014, PMID: 26196691

[ref141] Roscetto E. Bellavita R. Paolillo R. Merlino F. Molfetta N. Grieco P.. (2021). Antimicrobial activity of a Lipidated Temporin L analogue against Carbapenemase-producing *Klebsiella pneumoniae* clinical isolates. Antibiotics 10:1312. doi: 10.3390/antibiotics10111312, PMID: 34827250 PMC8614721

[ref142] Roscetto E. Contursi P. Vollaro A. Fusco S. Notomista E. Catania M. R. (2018). Antifungal and anti-biofilm activity of the first cryptic antimicrobial peptide from an archaeal protein against Candida spp. clinical isolates. Sci. Rep. 8:17570. doi: 10.1038/s41598-018-35530-0, PMID: 30514888 PMC6279838

[ref143] Ryder K. Bekhit A. E.-D. McConnell M. Carne A. (2016). Towards generation of bioactive peptides from meat industry waste proteins: generation of peptides using commercial microbial proteases. Food Chem. 208, 42–50. doi: 10.1016/j.foodchem.2016.03.121, PMID: 27132822

[ref144] Saito T. Kawabata S. Shigenaga T. Takayenoki Y. Cho J. Nakajima H.. (1995). A novel big defensin identified in horseshoe crab hemocytes: isolation, amino acid sequence, and antibacterial activity. J. Biochem. 117, 1131–1137. doi: 10.1093/oxfordjournals.jbchem.a124818 8586631

[ref145] Scarsini M. Tomasinsig L. Arzese A. D'Este F. Oro D. Skerlavaj B. (2015). Antifungal activity of cathelicidin peptides against planktonic and biofilm cultures of Candida species isolated from vaginal infections. Peptides 71, 211–221. doi: 10.1016/j.peptides.2015.07.023, PMID: 26238597

[ref146] Segura A. Moreno M. Madueño F. Molina A. García-Olmedo F. (1999). Snakin-1, a peptide from potato that is active against plant pathogens. Mol. Plant-Microbe Interact. 12, 16–23. doi: 10.1094/MPMI.1999.12.1.16, PMID: 9885189

[ref147] Selsted M. E. Harwig S. S. Ganz T. Schilling J. W. Lehrer R. I. (1985). Primary structures of three human neutrophil defensins. J. Clin. Invest. 76, 1436–1439. doi: 10.1172/JCI112121, PMID: 4056036 PMC424095

[ref148] Sengupta J. Saha S. Khetan A. Sarkar S. K. Mandal S. M. (2012). Effects of lactoferricin B against keratitis-associated fungal biofilms. J. Infect. Chemother. 18, 698–703. doi: 10.1007/s10156-012-0398-3 22410856

[ref149] Sewczyk T. Hoog Antink M. Maas M. Kroll S. Beutel S. (2018). Flow rate dependent continuous hydrolysis of protein isolates. AMB Express 8:18. doi: 10.1186/s13568-018-0548-9, PMID: 29429128 PMC5812119

[ref150] Shams M. V. Nazarian-Firouzabadi F. Ismaili A. Shirzadian-Khorramabad R. (2019). Production of a recombinant dermaseptin peptide in *nicotiana tabacum* hairy roots with enhanced antimicrobial activity. Mol. Biotechnol. 61, 241–252. doi: 10.1007/s12033-019-00153-x, PMID: 30649664

[ref151] Sharma P. Chaudhary M. Khanna G. Rishi P. Kaur I. P. (2021). Envisaging antifungal potential of Histatin 5: a physiological salivary peptide. J. Fungi 7:1070. doi: 10.3390/jof7121070 PMC8707063 34947052

[ref152] Shishido T. K. Humisto A. Jokela J. Liu L. Wahlsten M. Tamrakar A.. (2015). Antifungal compounds from cyanobacteria. Mar. Drugs 13, 2124–2140. doi: 10.3390/md13042124, PMID: 25871291 PMC4413203

[ref153] Shrestha C. L. Oña I. Muthukrishnan S. Mew T. W. (2007). Chitinase levels in rice cultivars correlate with resistance to the sheath blight pathogen Rhizoctonia solani. Eur. J. Plant Pathol. 120, 69–77. doi: 10.1007/s10658-007-9199-4

[ref154] Sibel Akalin A. (2014). Dairy-derived antimicrobial peptides: action mechanisms, pharmaceutical uses and production proposals. Trends Food Sci. Technol. 36, 79–95. doi: 10.1016/j.tifs.2014.01.002

[ref155] Silva P. M. Gonçalves S. Santos N. C. (2014). Defensins: antifungal lessons from eukaryotes. Front. Microbiol. 5:97. doi: 10.3389/fmicb.2014.00097, PMID: 24688483 PMC3960590

[ref156] Silva P. I. Jr. Daffre S. Bulet P. (2000). Isolation and characterization of gomesin, an 18-residue cysteine-rich defense peptide from the spider *Acanthoscurria gomesiana* hemocytes with sequence similarities to horseshoe crab antimicrobial peptides of the tachyplesin family. J. Biol. Chem. 275, 33464–33470. doi: 10.1074/jbc.M001491200, PMID: 10942757

[ref157] Simmaco M. Mignogna G. Canofeni S. Miele R. Mangoni M. L. Barra D. (1996). Temporins, antimicrobial peptides from the European red frog *Rana temporaria*. Eur. J. Biochem. 242, 788–792. doi: 10.1111/j.1432-1033.1996.0788r.x, PMID: 9022710

[ref158] Simon A. Kullberg B. J. Tripet B. Boerman O. C. Zeeuwen P. van der Ven-Jongekrijg J.. (2008). Drosomycin-like defensin, a human homologue of *Drosophila melanogaster* drosomycin with antifungal activity. Antimicrob. Agents Chemother. 52, 1407–1412. doi: 10.1128/AAC.00155-07, PMID: 18212107 PMC2292511

[ref159] Singh A. Upadhyay V. Upadhyay A. K. Singh S. M. Panda A. K. (2015). Protein recovery from inclusion bodies of *Escherichia coli* using mild solubilization process. Microb. Cell Factories 14:41. doi: 10.1186/s12934-015-0222-8, PMID: 25889252 PMC4379949

[ref160] Skerlavaj B. Benincasa M. Risso A. Zanetti M. Gennaro R. (1999). SMAP-29: a potent antibacterial and antifungal peptide from sheep leukocytes. FEBS Lett. 463, 58–62. doi: 10.1016/s0014-5793(99)01600-2, PMID: 10601638

[ref161] Slightom J. L. Metzger B. P. Luu H. T. Elhammer A. P. (2009). Cloning and molecular characterization of the gene encoding the Aureobasidin a biosynthesis complex in Aureobasidium pullulans BP-1938. Gene 431, 67–79. doi: 10.1016/j.gene.2008.11.011, PMID: 19084058

[ref162] Soltani S. Keymanesh K. Sardari S. (2007). In silico analysis of antifungal peptides. Expert Opin. Drug Discov. 2, 837–847. doi: 10.1517/17460441.2.6.837, PMID: 23489001

[ref163] Soravia E. Martini G. Zasloff M. (1988). Antimicrobial properties of peptides from Xenopus granular gland secretions. FEBS Lett. 228, 337–340. doi: 10.1016/0014-5793(88)80027-9 3125066

[ref164] Souza A. L. Díaz-Dellavalle P. Cabrera A. Larrañaga P. Dalla-Rizza M. De-Simone S. G. (2013). Antimicrobial activity of pleurocidin is retained in Plc-2, a C-terminal 12-amino acid fragment. Peptides 45, 78–84. doi: 10.1016/j.peptides.2013.03.030, PMID: 23603258

[ref165] Souza B. M. Mendes M. A. Santos L. D. Marques M. R. César L. M. M. Almeida R. N. A.. (2005). Structural and functional characterization of two novel peptide toxins isolated from the venom of the social wasp Polybia Paulista. Peptides 26, 2157–2164. doi: 10.1016/j.peptides.2005.04.026, PMID: 16129513

[ref166] Steckbeck J. D. Deslouches B. Montelaro R. C. (2014). Antimicrobial peptides: new drugs for bad bugs? Expert. Opin. Biol. Ther. 14, 11–14. doi: 10.1517/14712598.2013.844227, PMID: 24206062 PMC4109705

[ref167] Sung W. S. Park S. H. Lee D. G. (2008). Antimicrobial effect and membrane-active mechanism of Urechistachykinins, neuropeptides derived from *Urechis unicinctus*. FEBS Lett. 582, 2463–2466. doi: 10.1016/j.febslet.2008.06.015, PMID: 18570895

[ref168] Tam J. P. Wang S. Wong K. H. Tan W. L. (2015). Antimicrobial peptides from plants. Pharmaceuticals 8, 711–757. doi: 10.3390/ph8040711, PMID: 26580629 PMC4695807

[ref169] Tassin S. Broekaert W. F. Marion D. Acland D. P. Ptak M. Vovelle F.. (1998). Solution structure of ace-AMP1, a potent antimicrobial protein extracted from onion seeds. Structural analogies with plant nonspecific lipid transfer proteins. Biochemistry 37, 3623–3637. doi: 10.1021/bi9723515, PMID: 9521681

[ref170] Terras F. R. Torrekens S. Van Leuven F.. (1993). A new family of basic cysteine-rich plant antifungal proteins from Brassicaceae species. FEBS Lett. 316, 233–240. doi: 10.1016/0014-5793(93)81299-f, PMID: 8422949

[ref171] Theis T. Marx F. Salvenmoser W. Stahl U. Meyer V. (2005). New insights into the target site and mode of action of the antifungal protein of aspergillus giganteus. Res. Microbiol. 156, 47–56. doi: 10.1016/j.resmic.2004.08.006, PMID: 15636747

[ref172] Theis T. Stahl U. (2004). Antifungal proteins: targets, mechanisms and prospective applications. CMLS 61, 437–455. doi: 10.1007/s00018-003-3231-4, PMID: 14999404 PMC11146029

[ref173] Thery T. Lynch K. M. Arendt E. K. (2019). Natural antifungal peptides/proteins as model for novel food preservatives. Compr. Rev. Food Sci. Food Saf. 18, 1327–1360. doi: 10.1111/1541-4337.12480, PMID: 33336909

[ref174] Thevissen K. Ferket K. K. François I. E. Cammue B. P. (2003). Interactions of antifungal plant defensins with fungal membrane components. Peptides 24, 1705–1712. doi: 10.1016/j.peptides.2003.09.014, PMID: 15019201

[ref175] Thevissen K. Warnecke D. C. François I. E. Leipelt M. Heinz E. Ott C.. (2004). Defensins from insects and plants interact with fungal glucosylceramides. J. Biol. Chem. 279, 3900–3905. doi: 10.1074/jbc.M311165200, PMID: 14604982

[ref176] Thorn H. I. Guruceaga X. Martin-Vicente A. Nywening A. V. Xie J. Ge W.. (2024). MOB-mediated regulation of septation initiation network (SIN) signaling is required for echinocandin-induced hyperseptation in *Aspergillus fumigatus*. mSphere 9:e0069523. doi: 10.1128/msphere.00695-23, PMID: 38349166 PMC10964416

[ref177] Tracanna V. de Jong A. Medema M. H. Kuipers O. P. (2017). Mining prokaryotes for antimicrobial compounds: from diversity to function. FEMS Microbiol. Rev. 41, 417–429. doi: 10.1093/femsre/fux014, PMID: 28402441

[ref178] Tran D. Tran P. A. Tang Y. Q. Yuan J. Cole T. Selsted M. E. (2002). Homodimeric theta-defensins from rhesus macaque leukocytes: isolation, synthesis, antimicrobial activities, and bacterial binding properties of the cyclic peptides. J. Biol. Chem. 277, 3079–3084. doi: 10.1074/jbc.M109117200, PMID: 11675394

[ref179] Turcu R. Patterson M. J. Omar S. (2009). Influence of sodium intake on amphotericin B-induced nephrotoxicity among extremely premature infants. Pediatr. Nephrol. 24, 497–505. doi: 10.1007/s00467-008-1050-4, PMID: 19082636

[ref180] Ubukata M. Uramoto M. Isono K. (1984). The structure of neopeptins, inhibitors of fungal cell wall biosynthesis. Tetrahedron Lett. 25, 423–426. doi: 10.1016/S0040-4039(00)99901-5

[ref181] Van Baarlen P. Legendre L. Kan J. Van A. L. (2007). Plant defence compounds against botrytis infection. Botrytis Biol. Pathol. Control 22, 143–161. doi: 10.1007/978-1-4020-2626-39

[ref182] Van den Bergh K. P. Proost P. Van Damme J. Coosemans J. Van Damme E. J. Peumans W. J. (2002). Five disulfide bridges stabilize a hevein-type antimicrobial peptide from the bark of spindle tree (*Euonymus europaeus* L.). FEBS Lett. 530, 181–185. doi: 10.1016/s0014-5793(02)03474-9, PMID: 12387889

[ref183] van der Weerden N. L. Bleackley M. R. Anderson M. A. (2013). Properties and mechanisms of action of naturally occurring antifungal peptides. Cellular Mol. Life Sci. 70, 3545–3570. doi: 10.1007/s00018-013-1260-1, PMID: 23381653 PMC11114075

[ref184] Van Dijck P. Sjollema J. Cammue B. P. Lagrou K. Berman J. d'Enfert C.. (2018). Methodologies for in vitro and in vivo evaluation of efficacy of antifungal and antibiofilm agents and surface coatings against fungal biofilms. Microbial Cell 5, 300–326. doi: 10.15698/mic2018.07.638 29992128 PMC6035839

[ref185] Vieira Gomes A. M. Souza Carmo T. Silva Carvalho L. Mendonça Bahia F. Parachin N. S. (2018). Comparison of yeasts as hosts for recombinant protein production. Microorganisms 6:38. doi: 10.3390/microorganisms6020038, PMID: 29710826 PMC6027275

[ref186] Vizioli J. Bulet P. Hoffmann J. A. Kafatos F. C. Müller H. M. Dimopoulos G. (2001). Gambicin: a novel immune responsive antimicrobial peptide from the malaria vector *Anopheles gambiae*. Proc. Natl. Acad. Sci. USA 98, 12630–12635. doi: 10.1073/pnas.221466798, PMID: 11606751 PMC60105

[ref187] Vriens K. Cammue B. P. A. Thevissen K. (2014). Antifungal plant defensins: mechanisms of action and production. Molecules 19, 12280–12303. doi: 10.3390/molecules190812280, PMID: 25153857 PMC6271847

[ref188] Wakabayashi H. Abe S. Okutomi T. Tansho S. Kawase K. Yamaguchi H. (1996). Cooperative anti-Candida effects of lactoferrin or its peptides in combination with azole antifungal agents. Microbiol. Immunol. 40, 821–825. doi: 10.1111/j.1348-0421.1996.tb01147.x 8985937

[ref189] Wang K. Dang W. Xie J. Zhu R. Sun M. Jia F.. (2015). Antimicrobial peptide protonectin disturbs the membrane integrity and induces ROS production in yeast cells. Biochim. Biophys. Acta 1848, 2365–2373. doi: 10.1016/j.bbamem.2015.07.008, PMID: 26209560

[ref190] Wang J. Qiang J. Li J. Wang D. (2024). Effect of high sodium ion level on the interaction of AmB with a cholesterol-rich phospholipid monolayer. Front. Mol. Biosci. 11:1405383. doi: 10.3389/fmolb.2024.1405383, PMID: 38784666 PMC11111911

[ref191] Wei G. X. Campagna A. N. Bobek L. A. (2007). Factors affecting antimicrobial activity of MUC7 12-mer, a human salivary mucin-derived peptide. Ann. Clin. Microbiol. Antimicrob. 6:14. doi: 10.1186/1476-0711-6-14, PMID: 17996119 PMC2211505

[ref192] Wilde C. G. Griffith J. E. Marra M. N. Snable J. L. Scott R. W. (1989). Purification and characterization of human neutrophil peptide 4, a novel member of the defensin family. J. Biol. Chem. 264, 11200–11203. doi: 10.1016/S0021-9258(18)60449-1, PMID: 2500436

[ref193] World Health Organization (2022). WHO fungal priority pathogens list to guide research, development and public health action. World Health Organization. Geneva: World Health Organization.

[ref194] World Health Organization (WHO) (1999). Basic food safety for health workers. Geneva: World Health Organization (WHO).

[ref195] Wu Y. He Y. Ge X. (2011). Functional characterization of the recombinant antimicrobial peptide Trx-ace-AMP1 and its application on the control of tomato early blight disease. Appl. Microbiol. Biotechnol. 90, 1303–1310. doi: 10.1007/s00253-011-3166-x, PMID: 21380518

[ref196] Yan P.-S. Song Y. Sakuno E. Nakajima H. Nakagawa H. Yabe K. (2004). Cyclo(l-Leucyl-l-prolyl) produced by *Achromobacter Xylosoxidans* inhibits aflatoxin production by aspergillus parasiticus. Appl. Environ. Microbiol. 70, 7466–7473. doi: 10.1128/AEM.70.12.7466-7473.2004, PMID: 15574949 PMC535151

[ref197] Yan J. Yuan S. S. Jiang L. L. Ye X. J. Ng T. B. Wu Z. J. (2015). Plant antifungal proteins and their applications in agriculture. Appl. Microbiol. Biotechnol. 99, 4961–4981. doi: 10.1007/s00253-015-6654-6 25971197

[ref198] Yang L. Harroun T. A. Weiss T. M. Ding L. Huang H. W. (2001). Barrel-stave model or toroidal model? A case study on melittin pores. Biophys. J. 81, 1475–1485. doi: 10.1016/S0006-3495(01)75802-X, PMID: 11509361 PMC1301626

[ref199] Yeaman M. R. Yount N. Y. (2003). Mechanisms of antimicrobial peptide action and resistance. Pharmacol. Rev. 55, 27–55. doi: 10.1124/pr.55.1.2 12615953

[ref200] Zasloff M. (1987). Magainins, a class of antimicrobial peptides from Xenopus skin: isolation, characterization of two active forms, and partial cDNA sequence of a precursor. Proc. Natl. Acad. Sci. USA 84, 5449–5453. doi: 10.1073/pnas.84.15.5449, PMID: 3299384 PMC298875

[ref201] Zhang D. Lu Y. Chen H. Wu C. Zhang H. Chen L.. (2020). Antifungal peptides produced by actinomycetes and their biological activities against plant diseases. J. Antibiot. 73, 265–282. doi: 10.1038/s41429-020-0287-4, PMID: 32123311

[ref202] Zhang Q. Y. Yan Z. B. Meng Y. M. Hong X. Y. Shao G. Ma J. J.. (2021). Antimicrobial peptides: mechanism of action, activity and clinical potential. Mil. Med. Res. 8:48. doi: 10.1186/s40779-021-00343-2, PMID: 34496967 PMC8425997

[ref203] Zhang L. M. Yang M. Zhou S. W. Zhang H. Feng Y. Shi L.. (2023). Blapstin, a diapause-specific peptide-like peptide from the Chinese medicinal beetle Blaps rhynchopetera. Has Antifungal Funct. Microbiol. Spectr. 11:e0308922. doi: 10.1128/spectrum.03089-22, PMID: 37140456 PMC10269622

[ref204] Zhou J. Kong L. Fang N. Mao B. Ai H. (2016). Synthesis and functional characterization of MAF-1A peptide derived from the larvae of housefly, *Musca domestica* (Diptera: Muscidae). J. Med. Entomol. 53, 1467–1472. doi: 10.1093/jme/tjw110 27838615

[ref205] Zottich U. Da Cunha M. Carvalho A. O.. (2013). An antifungal peptide from *Coffea canephora* seeds with sequence homology to glycine-rich proteins exerts membrane permeabilization and nuclear localization in fungi. Biochim. Biophys. Acta 1830, 3509–3516. doi: 10.1016/j.bbagen.2013.03.007, PMID: 23500079

